# Improvement of alfalfa forage quality and management through the down‐regulation of *MsFTa1*

**DOI:** 10.1111/pbi.13258

**Published:** 2019-10-13

**Authors:** Christian D. Lorenzo, Pedro García‐Gagliardi, Mariana S. Antonietti, Maximiliano Sánchez‐Lamas, Estefanía Mancini, Carlos A. Dezar, Martin Vazquez, Gerónimo Watson, Marcelo J. Yanovsky, Pablo D. Cerdán

**Affiliations:** ^1^ Fundación Instituto Leloir IIBBA‐CONICET Buenos Aires Argentina; ^2^ Instituto de Agrobiotecnología de Rosario (INDEAR) CONICET Rosario Argentina; ^3^ Facultad de Ciencias Exactas y Naturales Universidad de Buenos Aires Buenos Aires Argentina

**Keywords:** flowering, *FLOWERING LOCUS T*, forage improvement, *Medicago sativa*, photoperiod

## Abstract

Alfalfa (*Medicago sativa L*.) is one of the most important forage crops worldwide. As a perennial, alfalfa is cut several times each year. Farmers face a dilemma: if cut earlier, forage nutritive value is much higher but regrowth is affected and the longevity of the stand is severely compromised. On the other hand, if alfalfa is cut later at full flower, stands persist longer and more biomass may be harvested, but the nutritive value diminishes. Alfalfa is a strict long‐day plant. We reasoned that by manipulating the response to photoperiod, we could delay flowering to improve forage quality and widen each harvesting window, facilitating management. With this aim, we functionally characterized the *FLOWERING LOCUS T* family of genes, represented by five members: *MsFTa1*,* MsFTa2*,* MsFTb1*,* MsFTb2* and *MsFTc*. The expression of *MsFTa1* correlated with photoperiodic flowering and its down‐regulation led to severe delayed flowering. Altogether, with late flowering, low expression of *MsFTa1* led to changes in plant architecture resulting in increased leaf to stem biomass ratios and forage digestibility. By manipulating photoperiodic flowering, we were able to improve the quality of alfalfa forage and management, which may allow farmers to cut alfalfa of high nutritive value without compromising stand persistence.

## Introduction

Alfalfa, *Medicago sativa L*, is regarded as the most important forage crop in the world (Putnam *et al*., 2001) with a global hay market in 2017 of 8.3 million metric tons (ITC, 2018). Such importance derives from the high quality of its forage, including the high content of protein, and therefore, it is used for dairy and meat production (Ball *et al*., 2001; Putnam *et al*., 2001). Other agronomic traits are highly desirable. Besides forage quality, alfalfa shows adaptability to different environments, abundant biomass yield, drought tolerance and, more important, capacity to fix nitrogen through symbiosis with rhizobia (Lei *et al*., 2017; Putnam *et al*., 2001; Radovic *et al*., 2009; Singer *et al*., 2018). The latter accounts for most of the N2 requirements of alfalfa and ensures the high protein levels in leaves (Bickoff *et al*., 1972; Heichel, 1983). As a deep‐rooted plant, alfalfa has an improved efficiency of water usage (Putnam *et al*., 2001) and nutrient uptake; together with N2 fixation, these are excellent qualities for sustainable agriculture (Kulkarni *et al*., 2018).

Despite its importance, the improvement of alfalfa lies behind other species of economic importance, for several reasons. First, it is an allogamous autotetraploid (Stanford, 1951), with 2n = 4x = 32 (Armstrong, 1954). Second, alfalfa is highly sensitive to inbreeding depression, which presumably occurs due to the accumulation of deleterious alleles while increasing homozygosity (Willis, 1999). Third, most cultivars are synthetic, which means they are produced by recurrent phenotypic selection after a few generations of panmictic crosses (Goodman, 2004), and therefore, high variability is found in commercial cultivars (Flajoulot *et al*., 2005). In this context, genetic engineering might play an important role in alfalfa improvement.

High‐quality forage production for use as cattle feed is the central cornerstone for livestock and dairy industries. Forage quality is dependent on various factors such as palatability, digestibility and the final animal performance (Ball *et al*., 2001). Digestibility is determined by the capacity of cattle to digest and metabolize the nutrient components provided by their feed (Adesogan *et al*., 2009; Flores‐Mar *et al*., 2017). Therefore, good quality fodder is characterized by high nutrient levels, a superior energy intake by the animal, elevated protein content (Allen, 1996; Ball *et al*., 2001) and low amount of non‐digestible components such as cellulose or lignin (Hatfield *et al*., 1994, 1999). Consequently, both yield and quality are the main variables aimed for improvement in forage crops.

Alfalfa is cut several times per year. Therefore, when to cut is an important decision since there is a trade‐off: as alfalfa matures, yield increases, but forage quality decreases. Further, if cuts are frequent, then persistence of the stand may be hampered, as regrowth is affected due to low level of accumulated reserves in the crown (Orloff, 2007). To aid in management decisions, alfalfa researchers have developed models that correlate alfalfa forage quality with easy‐to‐measure maturity parameters (Fick *et al*., 1988; Fick and Mueller, 1989; Orloff, 2007). For most farmers, up to a 10% blooming stage in the field is commonly considered a good maturity time predictor for high biomass and quality harvest (Bosworth and Stringer, 1992). This is so because after flowering onset, there is a major reallocation of photosynthetic resources from leaves to the new reproductive structures (Ruan *et al*., 2012). Additionally, transition to flowering comes alongside an increase in tissue lignification (Wang *et al*., 2013) and an increase in the ratio of stem to leaf tissue; the quality of stem tissue decreases with maturity whereas the quality of leaf tissue is relatively more stable (Fick *et al*., 1988; Kalu and Fick, 1981). The losses of forage quality can reach about 45% in relative feed value upon flowering (Bosworth and Stringer, 1992). It is calculated that a 1% loss in forage digestibility leads to a 3% loss in daily gains of beef cattle (Casler and Vogel, 1999). Therefore, we hypothesized that by delaying flowering, alfalfa could be improved in two aspects: first, to maintain high forage quality for longer periods and second, to facilitate management by extending the window of time farmers may harvest without severe losses in forage quality. Targeting flowering onset as a strategy for crop improvement has been performed in several species. In some crops like *Zea mays*,* Oryza sativa* or *Citrus sinensis,* an early flowering phenotype has been selected either during domestication or by conventional breeding (Fujino *et al*., 2013; Hefny, 2011; Hung *et al*., 2012; Velazquez *et al*., 2016). In other cases like lettuce (*Lactuca Sativa*) or alfalfa itself, research has been performed to develop strategies that favour a late transition to reproductive stages, since the process of flowering, as mentioned earlier, has a negative impact on biomass quality (Aung *et al*., 2015; Chen *et al*., 2017; Gao *et al*., 2016; Jung *et al*., 2016).

Flowering onset is a complex developmental transition that can be triggered by several pathways (Amasino, 2010). Both external and internal signals mediate this transition such as day length, temperature, light quality and plant age among others (Cerdan and Chory, 2003; Hyun *et al*., 2017; Lorenzo *et al*., 2016). Our aim was to identify a pathway that could be modified to delay flowering to the extent it improves forage quality and facilitates forage management but nonetheless allows flowering and adequate seed production. In alfalfa, both photoperiod and temperature are the most important stimuli to induce flowering and their effects are strongly cultivar dependent (Major *et al*., 1991; Nittler and Gibbs, 1959; Pearson and Hunt, 1972); it is a long‐day (LD) plant, which requires a minimum photoperiod of 12–14 h to effectively induce flowering (Major *et al*., 1991). Although the most important external cues that induce flowering in alfalfa have been characterized, the molecular gene network downstream is poorly understood. In model species such as *Arabidospis thaliana*, the central components of the photoperiod pathway are *FLOWERING LOCUS T (FT*) and *CONSTANS* (*CO*) (Corbesier *et al*., 2007; Lin *et al*., 2007). *FT* is expressed in phloem tissue, where it is up‐regulated by *CO* in response to long days (An *et al.,* 2004). When expressed, the small mobile FT protein travels from leaves to the meristem (Corbesier *et al*., 2007; Jaeger and Wigge, 2007; Mathieu *et al*., 2007) where it interacts with a B‐zip transcription factor known as FD (Abe *et al*., 2005; Wigge *et al*., 2005) forming a ‘florigen activation complex’ (FAC) (Taoka *et al*., 2011), which induces the expression of meristem identity genes such as *APETALA1*. More importantly, the roles of *FT*‐like genes are conserved in several species (Lifschitz *et al*., 2006; Lin *et al*., 2007; Tamaki *et al*., 2007) although some of them have acquired new functions, sometimes also related to photoperiod sensing (Hsu *et al*., 2006; Laurie *et al*., 2011; Lazakis *et al*., 2011; Lee *et al*., 2013; Navarro *et al*., 2011). However, no functional orthologues of *CO* have been identified in *Medicago truncatula*, suggesting that the photoperiod pathway may diverge in legumes, including alfalfa (Wong *et al*., 2014). In soybean (*Glycine max),* approximately 10 homologues of *FT* have been identified (Kong *et al*., 2010), with 6 of them having roles in flowering induction (Fan *et al*., 2014; Kong *et al*., 2010) and one as a flowering repressor (Zhai *et al*., 2014). In *Pisum sativum* and *Medicago truncatula,* five homologues of *FT* were identified (Hecht *et al*., 2011; Laurie *et al*., 2011) and, among them, only two are strong flowering inducers, suggesting that there may be additional subfunctionalization among the other *FT*‐like genes, as is the case in other species (Manoharan *et al*., 2016; Navarro *et al*., 2011).

With the objective of finding suitable candidate genes to delay flowering in alfalfa, we have functionally characterized the five homologues of *FT*,* MsFTa1*,* MsFTa2*,* MsFTb1*,* MsFTb2* and *MsFTc*. Among these, only *MsFTa1* showed a role as a flowering inductor. In our conditions, alfalfa plants never flowered in short days (SD), and plants that already flowered in long days (LD) rapidly reversed to a vegetative stage upon transfer to SD. Interestingly, such flowering behaviour correlated well with *MsFTa1* expression. We tested the roles of the five *msFT* genes in transgenic Arabidopsis, and, consistent with its photoperiod‐responsive patterns, *MsFTa1* was the most prominent flowering inductor. Finally, we down‐regulated the expression of *MsFTa1* in alfalfa and significantly delayed flowering in these transgenic lines. Later flowering led to an increase in the ratio of leaf to stem tissue and, more importantly, produced a significant increase in forage quality. Therefore, the manipulation of *MsFTa1* expression improves alfalfa in two ways: first, by increasing forage quality and second, by extending the window of time it may be harvested without significant quality losses.

## Results

### Photoperiodic induction of flowering in *Medicago sativa* cv Patricia

In order to study how day length induces flowering of alfalfa plants in our conditions, we tested the effects on flowering by growing plants in walk‐in incubators at 23°C in either LD or SD photoperiods of 150–250 μmoles/m^2^/s provided by LED neutral lightning (Figure S1). We selected specifically a LD of 16 h of light and 8 h of dark (16:8) and a SD of 8 h or light and 16 h of dark (8:16) because both conditions are representative of inductive and non‐inductive flowering photoperiods, respectively, in alfalfa (Major *et al*., 1991). Under LD conditions, alfalfa plants developed earlier and developed full flowers compared to SD conditions (Figure S1a), reaching reproductive stages in approximately 35 days (Figure S1b). Also, flowers appeared around the 14^th^ node for the primary axis, with secondary shoots flowering afterwards but at earlier nodes (Figure S1b). On the other hand, none of the plants grown under SD showed any sign of flowering induction during the whole assay. Further, we did not observe induced flowering in plants that were kept for 3 years in our SD conditions, suggesting that LD‐dependence of this cultivar of alfalfa is strict. Besides flowering time, day length also conditioned plant architecture, and plants grown in SD presented a bush‐like phenotype and also reduced height/node ratio (Figure S2), which is characteristic of alfalfa plants that grow at reduced rates under shorter photoperiods and lower temperatures (Fick *et al*., 1988).

### Functional characterization of *msFT* homologues

Both LD and warmer temperatures are important cues to induce flowering in alfalfa (Fick *et al*., 1988). Given that a role for *CO* is likely not conserved in alfalfa (Wong *et al*., 2014), but roles for *FT*‐like genes probably are, we choose this latter family for further investigation. More interestingly, *FT* genes integrate temperature and photoperiod signals in model systems (Song *et al*. 2013), turning these genes into ideal candidates for flowering time manipulation in alfalfa. Based on previous information from other legume species (Hecht *et al*., 2005, 2011; Laurie *et al*., 2011), we expected the conservation of 5 or more *FT* homologues in alfalfa. Searching the databases available in the *Medicago sativa Gene Idex* (O'Rourke *et al*., 2013) and in the Alfalfa Breeder's Toolbox (http://www.alfalfatoolbox.org), we were able to identify 5 putative *msFT*s. We performed a phylogenetic test aligning the sequences to other legume *FTs* (Figure S3). As a result (and though some of the branches resulted unsupported by the bootstrapping), out of the 5 *FTs*, we could preliminary associate 2 genes to the *FTa* legume clade (hence named *MsFTa1* and *MsFTa2*), two to the *FTb* clade (*MsFTb1* and *MsFTb2*) and one extra member belonging to the further distanced *FTc* clade (*MsFTc*). Additionally, we also aligned their amino acid sequences to the different sequences available in databases in order to search for different allelic variants (Figure S4), as alfalfa has been described to accumulate deleterious alleles (Willis, 1999).

**Figure 1:**
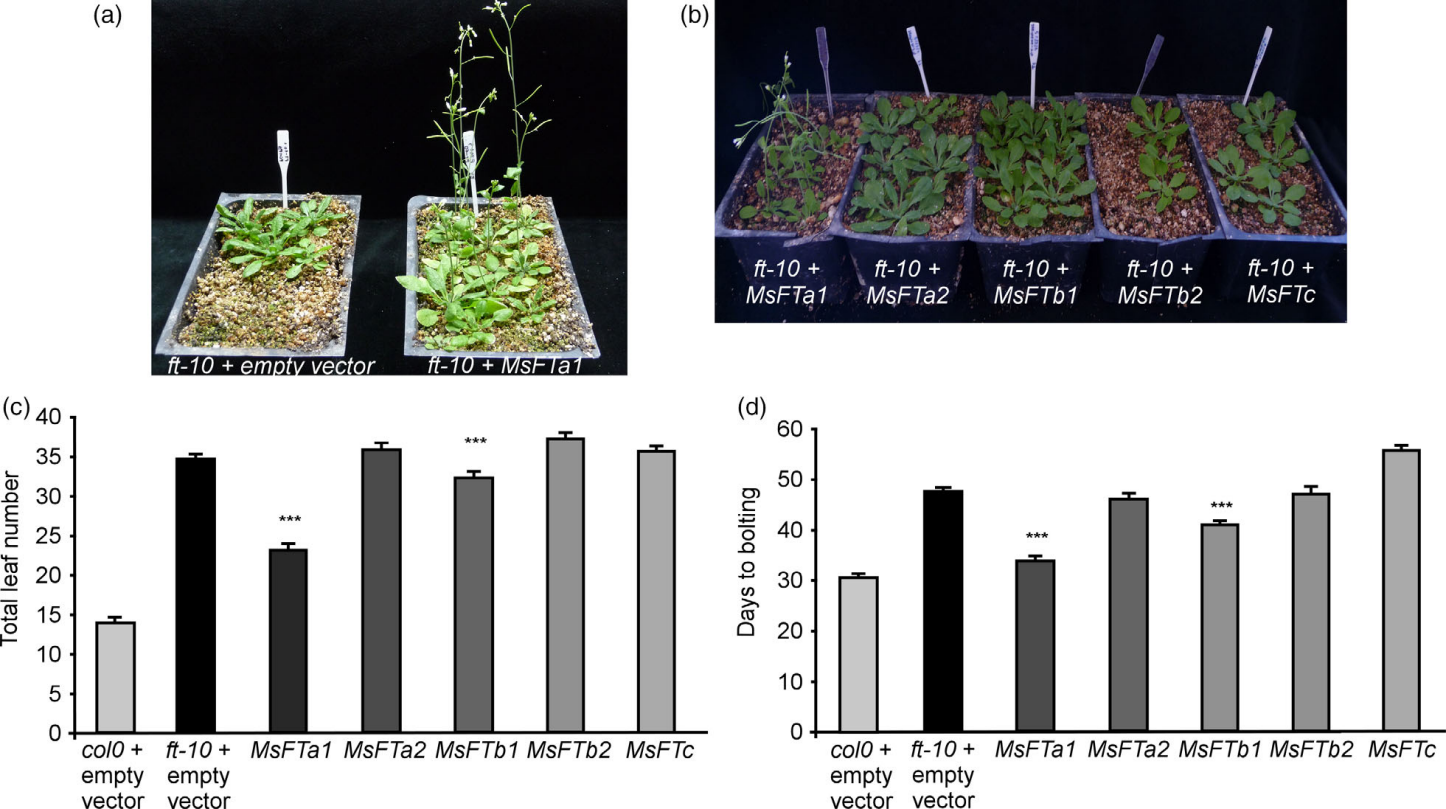
Constitutive expression of the 5 *msFTs* orthologues in *Arabidopsis thaliana*. (a) Early flowering of *ft‐10* lines transgenic for *35S::MsFTa1* compared to *ft‐10* controls transformed with the empty vector. (b) Flowering phenotypes of T1 transgenic lines bearing *35S::msFT* constructs as indicated. (c) Flowering time measured as both total leaf number and (d) days to floral bud appearance for *35S::MsFTa1*,* 35S::MsFTa2*,* 35S::MsFTb1*,* 35S::MsFTb2* and *35S::MsFTc* compared to *ft‐10* lines transformed with the empty vector. All plants were grown at 23°C under a LD photoperiod. Bars represent the means ± SE of at least 30 independent T1 plants per condition for lines in *ft‐10* background and at least 11 for WT background. Results were analysed by a one‐way ANOVA with posterior Dunnett test. Asterisks represent different levels of significance (*P* ≤ 0.05 = *, *P* ≤ 0.01 = **, *P* ≤ 0.001 = ***).

To test whether their roles in flowering are conserved in Arabidopsis, we cloned and expressed constitutively each of the five *msFTs* cDNAs in the late flowering *ft‐10* mutant of Arabidopsis (Figure 1) and in the WT background (Figure S6). Among the 5 genes, *MsFTa1* arose as a prominent flowering inductor compared to the other *msFTs*, with *MsFTb1* partially complementing the late flowering phenotypes of *ft‐10* mutants (Figure 1a,b). This is consistent with findings reported in *Medicago truncatula* (Laurie *et al*., 2011), but unlike these previous results, the *MsFTc* we identified had no effect in flowering induction in Arabidopsis (Figure 1c,d). Interestingly, our *MsFTc* presented a Glu 81 to Gly substitution, which could account for the lack of effects in Arabidopsis, as this amino acid is conserved in other functionally validated FTc homologues in legumes (Figure S5).

The same results were observed in the WT background (Figure S6), and relative expression for each *MsFT* was assessed to verify expression, particularly in non‐flowering *MsFTOX* lines (Figure S7).

**Figure 2:**
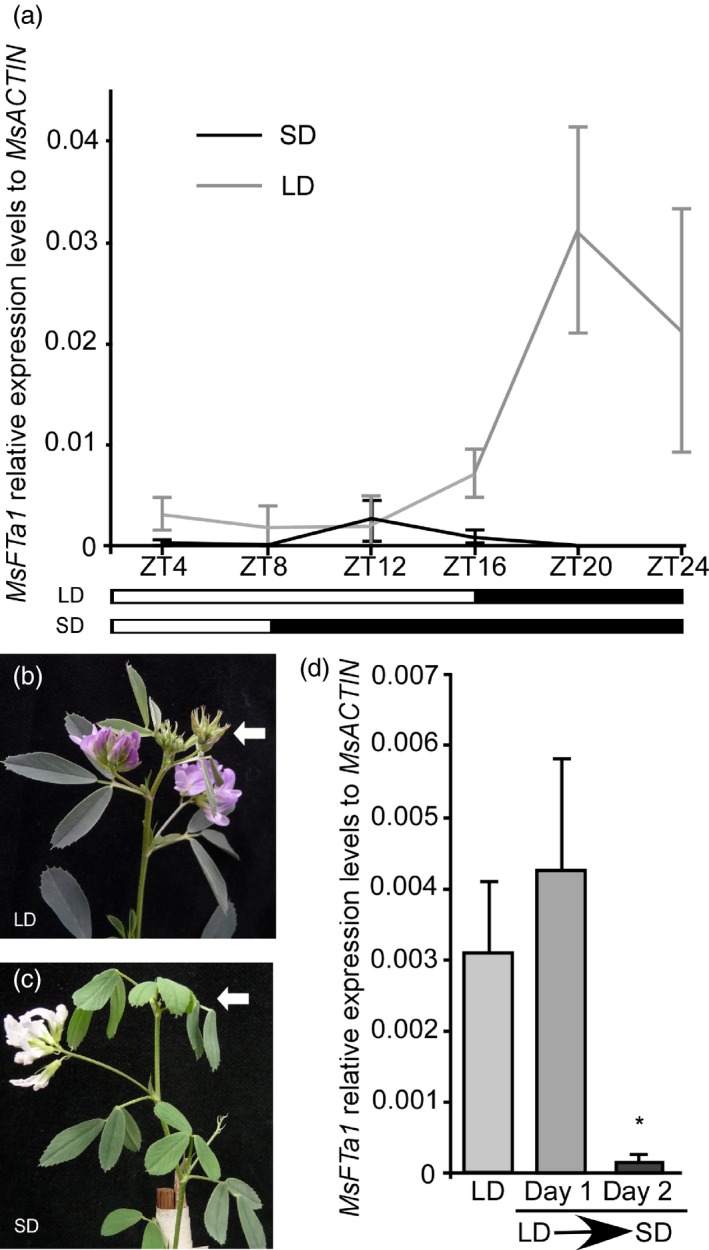
The expression of *MsFTa1* correlates with photoperiodic induction of flowering in alfalfa. (a) Trifoliated leaves of six‐week‐old alfalfa plants grown under either SD or LD photoperiods were harvested for RNA extraction. *MsFTa1* mRNA levels were measured by qPCR, relative to *msACTIN*. (b) Phenotype of a fully bloomed plant under LD and (c) phenotype of a plant transferred from LD to SD for one week. White arrows indicate the last fully developed apical bud. (d) Relative expression levels of *MsFTa1* (ZT16) in alfalfa plants that were grown either under LD, or grown under LD and transferred to SD for either 1 day or 2 days. Bars represent the means ± SE of 3 biological replicates per condition for (a) and 5 biological replicates for (d). Results were analysed by a one‐way ANOVA with posterior Dunnett test. Asterisks represent different levels of significance (*P* ≤ 0.05 = *).

If *MsFTa1* is involved in the flowering response to photoperiod, we would expect an increase in its expression under inductive photoperiods. Therefore, we performed a time course assay for plants grown under both SD and LD (Figure 2). Under LD, *MsFTa1* expression increased after ZT12 and peaked at ZT20. By the contrary, under SD *MsFTa1* only showed a mild peak around ZT12 (Figure 2a). Interestingly, plants that flowered in LD conditions reverted to a vegetative stage immediately after passage to SD conditions (Figure 2b,c), something evidenced by the appearance of a full vegetative node after the flowering node induced by the former condition. The flowers that completed development under SD were white (Figure 2c). These traits were previously reported with some cultivars (Fick *et al*., 1988). Based on the reversion of flowering upon return to SD conditions, we analysed *MsFTa1* expression at ZT16 in plants shifted from LD to SD conditions after 1 or 2 days (Figure 2d). We found that *MsFTa1* expression was significantly reduced after just two days of exposure to SD conditions. Altogether, these experiments show that *MsFTa1* expression correlates with flowering.

### Down‐regulation of *MsFTa1* delays flowering in alfalfa

To test the role of *MsFTa1* in the photoperiodic flowering of alfalfa, we decided to down‐regulate its expression. We designed an amiRNA complementary to *MsFTa1* following the procedures detailed by Weigel and col (Ossowski *et al*., 2008), using the microRNA319 of Arabidopsis as a backbone. Before transforming alfalfa, we proved the functionality of the amiRNA against *MsFTa1* by transforming the construct bearing the amiRNA (Figure S8) in Arabidopsis *ft‐10 MsFTa1*‐OX plants. Plants transformed with the *35S::amiRNA‐FTa1* construct displayed a late flowering phenotype (Figure S9a), measured as both total leaf number (Figure S9b) and days to bolting (Figure S9c). The delay in flowering correlated with a significant reduction in *MsFTa1* expression (Figure S9d), which restored the late flowering phenotype of the *ft‐10* mutant background.

**Figure 3:**
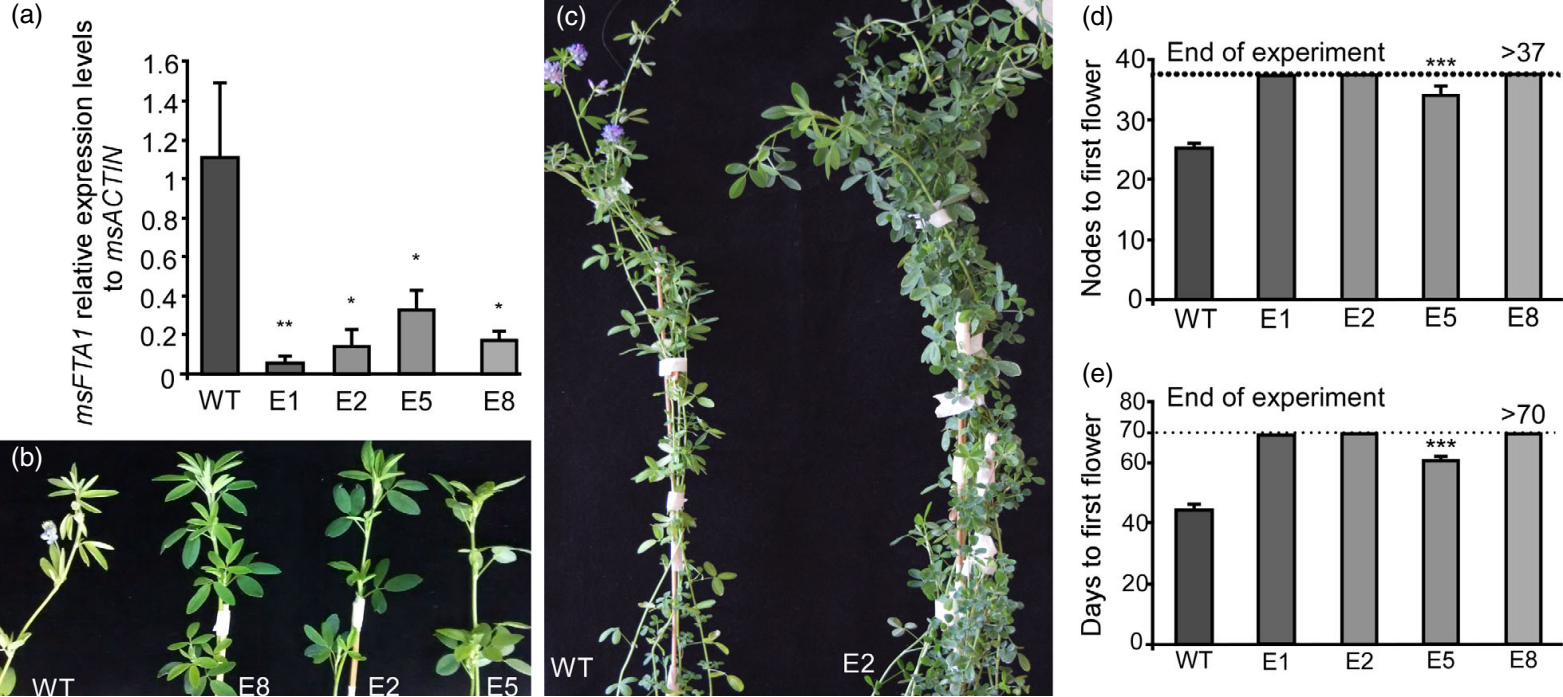
Down‐regulation of *MsFTa1* delays flowering in transgenic alfalfa. (a) Relative expression levels of *MsFTa1*. Leaves of 8‐week‐old plants grown under LD were harvested at ZT16 for RNA extraction. *MsFTa1* expression was quantified relative to *msACTIN* by qPCR for 4 independent transgenic lines and compared to WT controls. (b) Apices of 3 transgenic lines (E2, E5, E8) compared to WT regenerated controls. (c) Phenotype of a WT flowering plant compared to non‐flowering transgenic E2 line. (d) Flowering time measured as both nodes to first flower and (e) days to first flower. All plants were grown at 23°C under a LD photoperiod. Bars represent the means ± SE of 3 biological replicates for qPCR experiments and 8–10 individual grown clonal plants per line per condition for flowering assays. Results were analysed by a one‐way ANOVA with posterior Dunnett test. Asterisks represent different levels of significance (*P* ≤ 0.05 = *, *P* ≤ 0.01 = **, *P* ≤ 0.001 = ***).

After confirming the effectiveness of the *35S::amiRNA‐FTa1* construct in Arabidopsis, we generated 10 independent transgenic lines of alfalfa for this same construct (E1 to E10), and further analysed 4 of them (E1, E2, E5 and E8). The four lines showed reduced *MsFTa1* expression (Figure 3a), and therefore, we tested them for flowering time in LD conditions (Figure 3b‐e). All the transgenic lines showed delayed flowering compared to the regenerated controls, measured as both nodes to flowering and days to first flower appearance (Figure 3d,e). Out of the four independent lines, only the transgenic line E5 flowered during this experiment, while lines E1, E2 and E8 did not show any sign of upcoming flowering at their apical meristems by the end of the assay. Additionally, transgenic lines developed normally, as the total number of nodes per plant at the end of the assay was equal or even higher compared to WT plants (Figure S10a), and no differences were registered when analysing root dry weight (Figure S10b).

Due to the severe late flowering phenotype observed, we performed the same assay for an extended period of time in order to allow transgenic lines to flower (Figure S11). We found out that once again, transgenic lines E1, E2 and E8 did not flower (Figure S11a,b) consistently with our previous data. We did observe though, the occasional appearance of flowers in some plants, that reverted back to vegetative nodes almost immediately. This suggests that the levels of *FTA1* expression were not sufficient to induce robust flowering in these conditions.

### Down‐regulation of *MsFTa1* leads to changes in plant architecture and biomass partitioning

**Figure 4:**
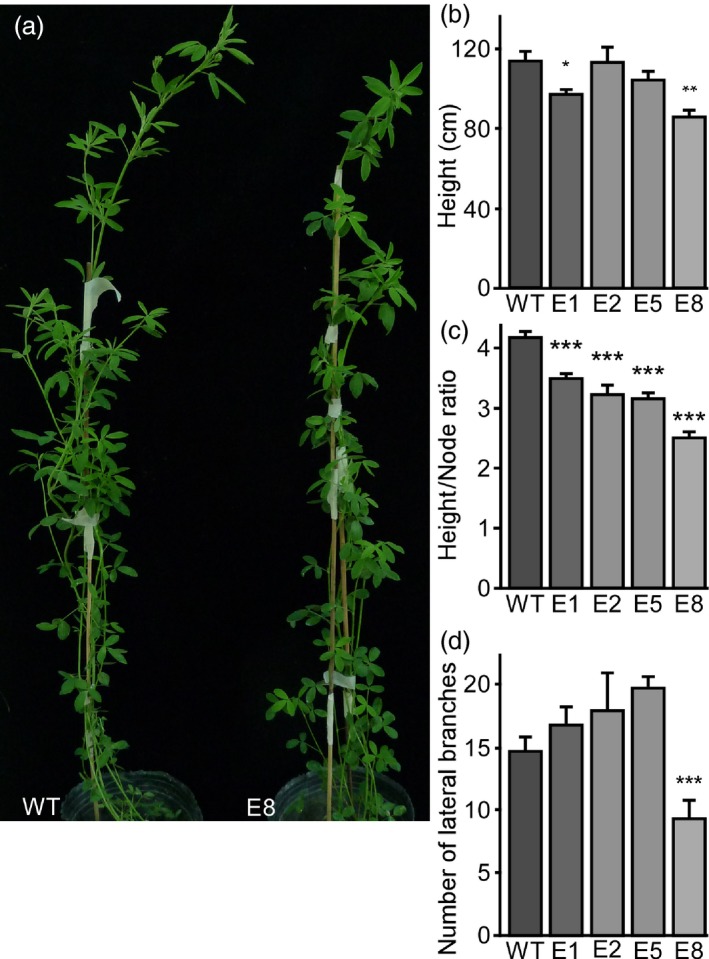
Down‐regulation of *MsFTa1* alters plant architecture. (a) Phenotype of a WT plant compared to a transgenic E8 line. (b) Height, (c) height to node ratio and (d) number of lateral branches of the indicated transgenic lines compared with the WT regenerated lines. Measurements were taken after two months of growth at 23°C under a LD photoperiod. Bars represent the means ± SE of 8–10 individual grown clonal plants per line. Results were analysed by a one‐way ANOVA with posterior Dunnett test. Asterisks represent different levels of significance (*P* ≤ 0.05 = *, *P* ≤ 0.01 = **, *P* ≤ 0.001 = ***).

Since flowering and non‐flowering plants differ in architecture (Figure S2), we analysed how transgenic lines with delayed flowering developed under LD conditions (Figure 4). Transgenic amiRNA lines had reduced height and reduced height/node ratios compared to WT plants (Figure 4a–c), which most likely resulted from decreased internode length. Additionally, the number of lateral branches did not differ from controls, except for line E8, which showed a reduced number of lateral branches (Figure 4d). These differences, though, were not observed in extended time assays after 4 months (Figure S11c,d)

**Figure 5:**
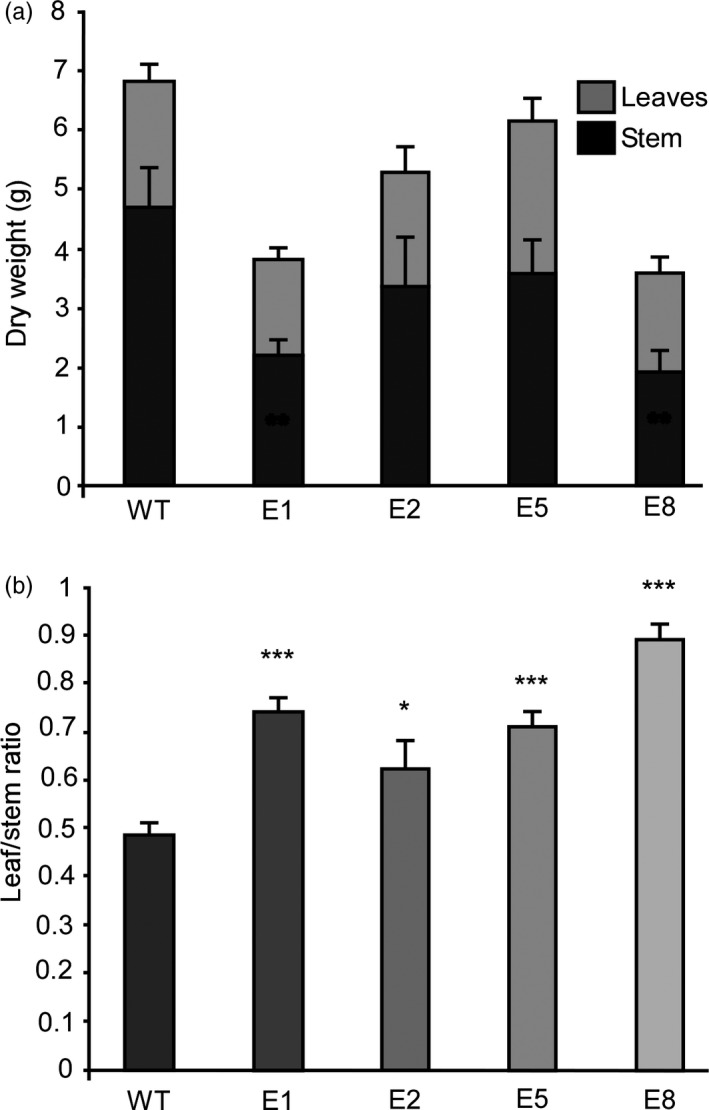
Down‐regulation of *MsFTa1* improves the ratio of leaf to stem biomass (a) Total dry weight partitioned as leaf dry weight (light grey bars) and stem dry weight (dark grey bars) and (b) leaf / stem ratio for 4 transgenic lines (E1, E2, E5, E8) overexpressing *amiRNA‐FTA1* compared to WT regenerated controls. All dry weights were determined two weeks after the last WT plant flowered. All plants were grown at 23°C under a LD photoperiod. Bars represent the means ± SE of 8–10 individual grown clonal plants per line. Results were analysed by a one‐way ANOVA with posterior Dunnett test. Asterisks represent different levels of significance (*P* ≤ 0.05 = *, *P* ≤ 0.01 = **, *P* ≤ 0.001 = ***).

Leaf biomass is of higher quality than stem biomass (Popovic *et al*., 2001; Wang and Kinsella, 1975), and therefore, we studied the biomass accumulation in stems and leaves (Figure 5). We grew the plants under LD until the controls reached full flowering. Leaf tissue and stems were separately collected from each plant and dried. Total dry weight diminished in transgenic lines at the expense of stem tissue (Figure 5a), while biomass accumulation in leaves remained unaffected. This led to a significant increase in the leaf/stem ratio, which is an important estimator of forage quality (Figure 5b). Similar results were obtained in extended assays at 120 days (Figure S12).

### Down‐regulation of *MsFTa1* leads to an increase in forage quality

**Figure 6:**
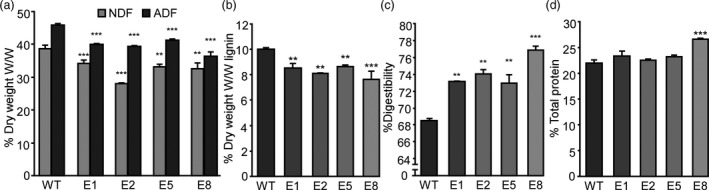
Down‐regulation of *MsFTa1* improves forage quality. (a) Percentage of NDF and ADF, (b) % of lignin, (c) % of digestibility and (d) % of total crude protein of samples belonging to 4 transgenic lines (E1, E2, E5, E8) overexpressing *amiRNA‐FTa1* compared to WT regenerated controls. Bars represent the means ± SE of 2–3 biological samples composed of 3–4 plants per line. Results were analysed by a one‐way ANOVA with posterior Dunnett test. Asterisks represent different levels of significance (*P* ≤ 0.05 = *, *P* ≤ 0.01 = **, *P* ≤ 0.001 = ***).

As a decrease in non‐digestible and lignified tissue is a desirable trait for alfalfa biomass (Ball *et al*., 2001), and since the transition to flowering is accompanied by a reduction in forage digestibility (Albrecht *et al*., 1987; Nordkvist and Åman, 1986), we assayed the forage quality of transgenic lines with delayed flowering and compared them to WT controls (Figure 6). Transgenic lines showed a significant decrease in both neutral detergent fibre (NDF) and acid detergent fibre (ADF) (Figure 6a) and a reduction in lignin content (Figure 6b), which is consistent with the observed increase in digestibility (Figure 6c). Also, some lines presented a significant increase in crude protein levels (Figure 6d), something that may derive from additional leaf biomass

### Plant architecture and late flowering are consistent through multiple cut and growth cycles

Since alfalfa biomass harvest usually involves multiple cut and regrowth cycles throughout the year, we wanted to confirm whether the late flowering phenotype and the changes in plant architecture observed at first cut stage were maintained in subsequent regrowth periods. Also, this late flowering trait might be compromised as the plant ages, since there is a chance that the age‐related pathway (Aung *et al*., 2015; Gao *et al*., 2016, 2018) may bypass photoperiod dependency.

**Figure 7:**
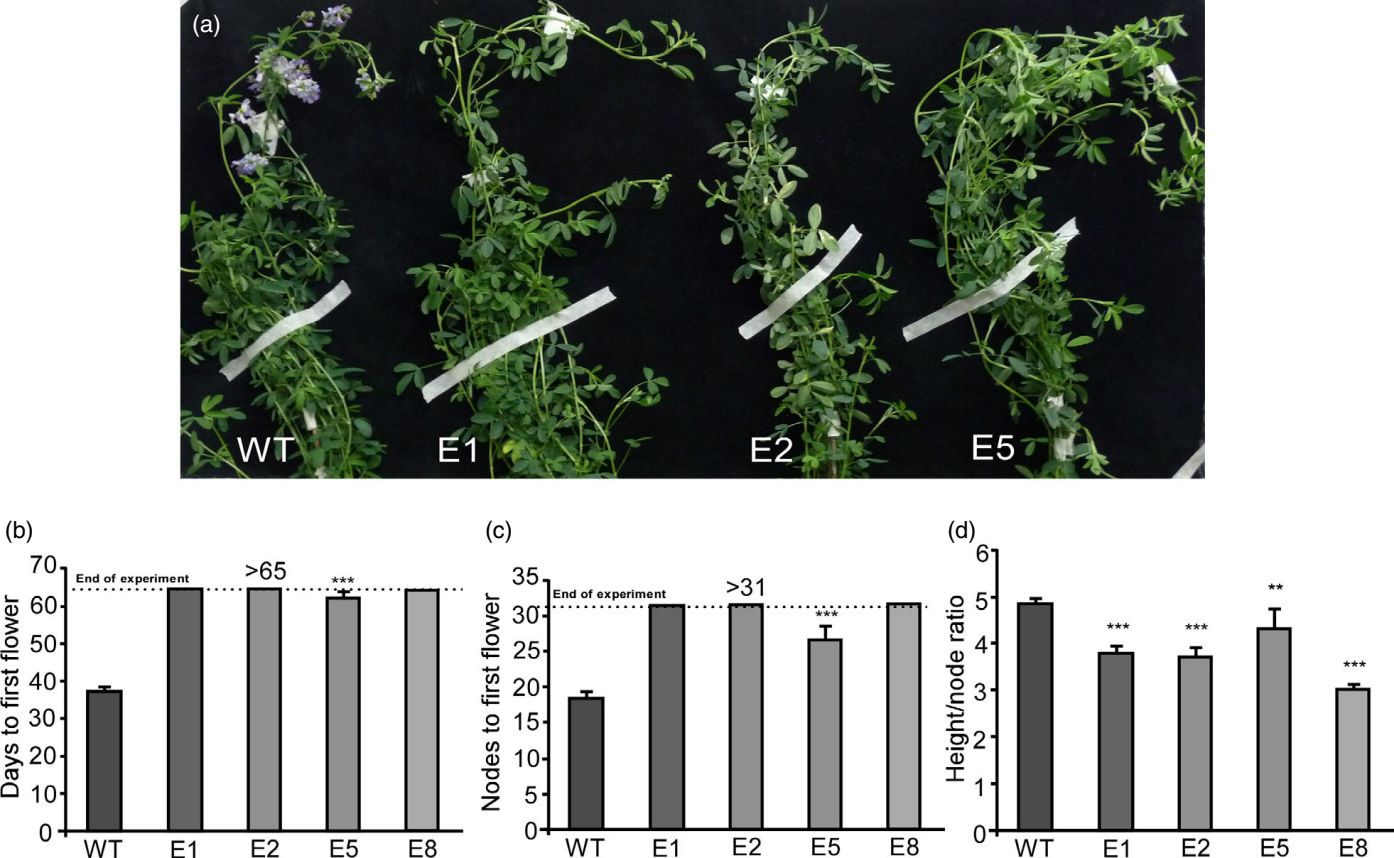
Late flowering and plant architecture phenotypes of *amiRNA‐FTa1* transgenic lines are stable after subsequent cut and regrowth cycles. (a) Phenotype of WT plants compared to transgenic lines after cut and a second regrowth cycle. (b) Days to first flower, (c) nodes to first flower and (d) height / node ratio of transgenic lines after cut and a second regrowth cycle. All plants were grown at 23°C under a LD photoperiod. Bars represent the means ± SE of 8–10 individual grown clonal plants per line. Results were analysed by a one‐way ANOVA with posterior Dunnett test. Asterisks represent different levels of significance (*P* ≤ 0.05 = *, *P* ≤ 0.01 = **, *P* ≤ 0.001 = ***).

Therefore, we tested the flowering time of *amiRNAFTa1* lines after a regrowth cycle under the same inductive flowering conditions previously assayed (Figure 7). As observed with the assays after the first cut (Figure 3), all transgenic lines showed a delayed flowering phenotype, measured as both days to first flower and node number (Figure 7a–c). Once again, the E5 line flowered under these conditions, while lines E1, E2 and E8 did not, therefore indicating a clear conservation of the same flowering phenotypes. Additionally, we observed similar decreases in the height/node ratio (Figure 7d). These experiments show that the late flowering phenotypes are stable through subsequent cut and regrowth periods and underscore the potential of transgenic alfalfa *amiRNA*‐*FTa1* lines for use in the field.

## Discussion

Regulation of flowering time is an important agronomic trait for several grain and forage crops (Blumel *et al*., 2015). The decrease in forage quality observed at flowering stages is a general problem with forage crops, and it occurs in grasses and in legumes. The impact in productivity is high. It is calculated that for each 1% increase in digestibility, the daily gains of beef cattle increase by 3% (Casler and Vogel, 1999). Here, we devised a strategy to improve the management and forage quality of alfalfa by manipulating the time to flowering. This strategy has two advantages. First, it delays flowering maintaining higher forage quality for longer periods, and second, it improves management by allowing later harvesting during the several cut and growth cycles.

We reasoned that a strategy devised to manipulate the photoperiod pathway would have reduced penalties in other agronomic traits. In our assays, flowering was strictly dependent on LD photoperiods. As the *CO*‐like genes are not conserved in alfalfa, we focused in the *FT* family of genes. We identified *MsFTa1, MsFTa2, MsFTb1, MsFTb2 and MsFTc* which resulted similar to the *FT* family in *Medicago truncatula* (Laurie *et al*., 2011). We found that *MsFTc* did not promote flowering when ectopically expressed in Arabidopsis, in contrast to *Medicago truncatula mtFTC* and pea *psFTC* where said orthologues induced a very early bolting in Arabidopsis (Hecht *et al*., 2011; Laurie *et al*., 2011). We identified a change in Glu 81 for a Gly in our Patricia cultivar. This substitution is nearby Tyr 82, a well‐known conserved amino acid important for FT function (Hanzawa *et al*., 2005), and therefore could be affecting its function in Arabidopsis (Figure S5), although we cannot rule out that it may still be functional in alfalfa. It would be interesting to study in the future if this allele represents a flowering QTL in alfalfa.

Only *MsFTa1* and, to a lesser extent, *MsFTb1* were able to induce flowering in Arabidopsis (Figure 1), consistent with reports in *Medicago truncatula* (Laurie *et al*., 2011). However, *Medicago truncatula* is an annual species while alfalfa is a perennial. The molecular basis for iteroparity in alfalfa is unknown. Interestingly, we found that *MsFTa1* expression was up‐regulated at later hours of a LD photoperiod (Figure 2a). These results partially follow reports in *Medicago truncatula* (Laurie *et al*., 2011). Laurie *et al*. have shown that though *MtFTa1* mRNA was induced by LD, its expression remained high throughout the LD light phase, peaking at ZT0 and ZT8. In our assays, *MsFTa1* displayed robust cycling in alfalfa reaching its peak at ZT20. Further, the induction of *MsFTa1* expression was reversed after just two days of plants being returned to SD (Figure 2d), which could account for the reversal of flowering under the same conditions (Figure 2b,c). Notwithstanding, in *Medicago truncatula*, shifts of plants from LD to SD also reverse *MtFTa1* expression, although just three long days are enough to induce a commitment to flowering (Laurie *et al*., 2011). Therefore, the response of *MsFTa1* expression to LD to SD shifts might not be the only mechanism to explain iteroparity in alfalfa; knowing its basis requires further testing.

Consistent with its important role in flowering induction, silencing of *MsFTa1* in alfalfa resulted in a late flowering phenotype (Figure 3). This delay in flowering also came along with a reduction in height/node ratio and a shortening of internodes (Figure 4), partially mimicking the phenotypes we observed when growing WT plants under SD (Figure S2).

The delay of flowering time also had a strong effect over biomass allocation and distribution. As previously mentioned, alfalfa forage decays as plants transition from vegetative to reproductive stages, since tissue tends to lignify and there is a decrease in the leaf/stem ratios (Fick and Mueller, 1989; Hall et al., 1992; Kalu and Fick, 1983). By delaying flowering, we were able to modify plant architecture, obtaining high leaf/stem ratios, which were mostly due to a decrease in stem biomass (Figure 5). As expected, increased leaf/stem ratios also correlated with improved forage quality. Late flowering plants showed reduced levels of NDF, ADF and lignin (Figure 6a,b), and increased digestibility (Figure 6c). Digestibility increased by at least 4%, which may potentially improve animal weight gain by 12% (Casler and Vogel, 1999). Finally, the late flowering and plant architecture phenotypes were robust and consistent among the subsequent cycles of cut and regrowth (Figure 7). It is important to highlight that out of the 4 transgenic lines observed, only line E5 did fully flower during our assays (Figure 3 and Figure S11), while still improving forage quality (Figure 6). Intermediate phenotypes such as the one displayed by E5 line would be more appropriate for field trials, as seed production is also an important trait for alfalfa breeders.

Two successful strategies have been designed to improve both alfalfa yield and forage quality by biotechnological means. A promising approach was the overexpression of alfalfa microRNA156 (miRNA156); transgenic plants displayed increased total biomass, root length and delayed flowering (Aung *et al*., 2015). As miRNA156 has multiple targets in the *SPL* family, it may have pleiotropic effects (Gao *et al*., 2016). Therefore, the same authors down‐regulated *msSPL13* specifically and were able to delay flowering by one week in transgenic alfalfa (Gao *et al*., 2018). The second strategy was aimed at reducing lignin content, which has been effectively tackled by down‐regulating genes involved in the lignin biosynthetic pathway such as cinnamyl alcohol dehydrogenases (Baucher *et al*., 1999), hydroxycinnamoyl coenzyme A (Gallego‐Giraldo *et al*., 2011) and caffeoyl‐CoA 3‐O‐methyltransferase (CCoAOMT) (Guo *et al*., 2001a,b). The last approach proved particularly successful because it combined the silencing of a specific gene in the lignin biosynthetic pathway and employed an adequate promoter to ensure specific tissue down‐regulation, resulting in about 3% increase in digestibility and reaching a commercial product recently (Barros *et al*., 2019). Interestingly, this approach could be combined with ours to further increase forage digestibility.

Our approach was effective to improve forage quality and delay flowering. Field trials are on the way, and we hope they will help in defining the future steps to convert this technology in a useful product, improving the sustainability of agriculture and forage crop management.

## Experimental procedures

### Plant material and flowering time assays

For initial photoperiod assays, seeds of *Medicago sativa* cv Patricia (Fall dormancy 7) were provided by the Instituto Nacional de Tecnología Agropecuaria (INTA). Seeds were surface sterilized with ethanol 70% followed by SDS/sodium hypochlorite (1 min each), rinsed with sterile water, dried in a vertical flow cabinet and further treated with chlorine in vapour phase. Seeds were plated in 0,8% agar half strength Murashige and Skoog media (MS) (Murashige and Skoog, 1962) and stratified for 3 days in darkness at 4 °C. Seedlings were then transferred to individual 1.5‐L pots with a mixture of 3:1:1 soil, perlite and vermiculite supplemented with Red Hakaphos fertilizer (Compo Agricultura, http://www.compo.es), and placed under inductive flowering conditions of LD and 23 °C with a PAR of 150–250 μmoles/m^2^/s.

### Generation of transgenic alfalfa lines

Alfalfa plant transformation was performed by Instituto de Agrobiotecnologia de Rosario (INDEAR). Methodology was based on the protocol described by Samac *et al*. (Samac and Austin‐Phillips, 2006). Folioles and petioles from adult alfalfa plants (cultivar null 15 derived from cultivar C2‐3, defined now as WT) were excised and surface sterilized in a laminar flow hood by rinsing them in a 70% ethanol and 20% NaClO solution. Folioles were divided in half while petioles were severed to obtain 1 cm explants. Explants were then co‐cultivated with *Agrobacterium tumefaciens* LBA4404 strain harbouring the *amiRNA‐FTa1* construct. After 4‐5 days of co‐cultivation, explants were then transferred to MS medium with increasing concentrations of ammonium glufosinate. After selection, transformed callus were plated on Gamborg medium B5 supplemented with 2,4 D and Kinetin to obtain embriogenic calli. After 40 days, developed calli were transferred to MS medium without growth regulators for embryo inducement and germination. Transgenic resistant embryos were then grown into developed plants and after acclimation were transferred to soil in 1‐lt pots with a mixture of 3:1:1 soil, perlite and vermiculite.

### Plant assays with alfalfa

For flowering time determination, alfalfa transgenic lines and their controls were placed under non‐inductive flowering conditions (23 °C SD) for at least one month. Afterwards, stakes were made from stems, cutting below the fourth node, counting from meristem to base, and placed in grow mix and, under LD conditions until rooted, then transferred to individual 1.5‐L pots with a mixture of 3:1:1 soil, perlite and vermiculite, supplemented with Red Hakaphos fertilizer. Rooted stakes developed into plants were then placed under inductive flowering conditions of LD and 23 °C with a PAR of 150–250 μmoles/m^2^/s. Flowering time was determined by the appearance of the first flowering bud in each plant. The duration of experiments was of 70 days for initial assays and, additionally, for 120 days for extended time experiments. Days to flowering, number of nodes in the longest stem (Sachs, 1999), internode length and plant height were registered in each condition at the time of flowering. At the end of the experiments, stem, petiole and leaf tissue of each plant were dried at 50°C for 5 days for dry weight determinations. For root dry weight, transgenic and WT lines were grown in a mixture of soil and thin vermiculite in 3‐L pots. Total root dry weight was collected at full vegetative stages for WT and transgenic plants and dried at 50°C for 5 days.

### Phylogenetic analysis

Amino acid sequences of *msFTs* were obtained from the MSGI, and putative orthologues sequences from *Glycine max*,* Medicago truncatula*,* Lotus japonicus*,* Cajanus cajan, Cicer aerinethum* and *Arabidopsis thaliana* were obtained from GenBank databases. Sequences were analysed using MEGA (Tamura *et al*., 2011). Alignments were performed with MUSCLE (Edgar, 2004), and a phylogenetic tree was created using the maximum likelihood method with a bootstrapping of 1000. Sequences belonging to legumes were obtained from the legume IP website (http://plantgrn.noble.org/LegumeIP/).

### Cloning and ectopic expression of *msFTs* in *Arabidopsis*

Alfalfa *FT* homologues *MsFTa1, MsFTa2, MsFTb1, MsFTb2 and MsFTc* were PCR amplified from cDNA samples using primers bearing BamHI, SalI or XbaI restriction sites and cloned in a binary plasmid with these enzymes (New England Biolabs) under the 35S promoter. All constructs were checked by Sanger sequencing (Macrogen, Korea), transformed in *Agrobacterium tumefaciens* GV3101 and introduced into *Arabidopsis thaliana ft‐10* and *Col‐0* (defined as WT for Arabidopsis assays) backgrounds by floral dip transformation (Clough and Bent, 1998). Selection of transformants was performed by plating in MS media supplemented with ammonium glufosinate or kanamycin (Duchefa).

### Assembly, cloning and ectopic expression of *amiRNA‐FTa1*

A 21‐pb sequence belonging to *MsFTa1* (TTATTGAAATTTTGTCGCCAT) was selected employing the web tool WMD3 (http://wmd3.weigelworld.org/cgi-bin/webapp.cgi) as a suitable target sequence. The final amiRNA target sequence (Figure S8) was chosen for its minimal off targets and similar secondary structure to microRNA319. (http://unafold.rna.albany.edu/?q=mfold/RNA-Folding-Form). Primers and protocols to assemble the amiRNA were performed based on the procedures detailed in *wmd3.weigelworld.org/*. Once assembled, the microRNA was cloned into the pBluescript vector and then subcloned under the 35S promoter in a binary vector with ammonium glufosinate resistance. To evaluate its efficacy, the *amiRNA‐FTa1* construct was transformed in Agrobacterium and then by floral dip transformation (Clough and Bent, 1998) into Arabidopsis *ft‐10 MsFTa1* OX T3 lines and transformants selected for sulfadiazine – kanamycin and ammonium glufosinate resistance.

### qPCR measurements

Total RNA was extracted from trifoliate leaves at ZT 16 using TRIzol (ThermoFisher Scientific), and 1 ¼g was employed to generate oligo‐dT primed cDNAs by using MMLV reverse transcriptase (Life technologies). For PCR reactions, primers were designed using the respective sequences from the MSGI (Table S1). For qPCRs, cDNAs were amplified with Paq Hot Start DNA polymerase (Stratagene), and *msActin2* was used as reference transcript in alfalfa (Wang *et al*., 2015) and *UBQ10* as reference in Arabidopsis. All determinations were performed on a Roche 480 lightcycler, and calculations were done following the Livak method (Livak and Schmittgen, 2001).

### Determination of forage quality assays

For determination of forage quality, samples were analysed as detailed in Bertoia and Aulicino, 2014; (Bertoia and Aulicino, 2014). Whole plant biomass was collected after a second and regrowth cycle and was dried at 50°C for 3–4 days and then further grounded in a mill (Fritsch co., Germany). Near infrared reflectance spectroscopy (NIRS) was used for forage quality determinations using a NIRS 6500 foss (Foss NIRS systems inc, silver spring, MD, USA), to collect the spectra of the ground samples in mini‐dishes (100 mm × 60 mm). NIRS calibration equations were determined using a subset of alfalfa dry weight samples previously analysed by routine laboratory methods. A pepsin–cellulose enzymatic assay was used to determine in vitro dry matter digestibility. Samples were incubated in pepsin (in 0.1 M HCl, 39.5 °C) for 24 h and then in cellulase preparations from *Trichoderma viridae* at 39.5 °C for 48 h.

## Conflict of interest

Authors Carlos Dezar, Martin Vazquez and Geronimo Watson are current employees of INDEAR S.A. All other authors state that they do not have any conflict of interest.

## Author contributions

CDL, CAAD, MV, GW, MJY and PDC conceived and designed the experiments. CDL, PGG, MSA, MSL and EM performed the experiments. CDL, EM and PDC analysed the data. CDL and PDC wrote the paper.

## Supporting information


**Figure S1** Effect of photoperiodic induction of flowering in *Medicago sativa* cv Patricia.

**Figure S2** Development and plant architecture of alfalfa plants grown under SD vs LD.

**Figure S3** Phylogenetic trees of proteins coded by 5 identified msFT orthologues in *Medicago sativa* compared to other legume orthologues.

**Figure S4** Aminoacid alignment of MsFT protein sequences including different alleles available in public databases.

**Figure S5** Aminoacid alignment of functionally chacterised *FTc* orthologues in legumes.

**Figure S6** Flowering time of transgenic WT Arabidopsis constitutively expressing *msFTs* orthologues.

**Figure S7** Expression levels of alfalfa *MsFTs* in Arabidopsis *ft‐10* background.

**Figure S8** Secondary structure and sequence of *amiRNA‐FTa1*

**Figure S9** Evaluation of *amiRNA‐FTa1* in *Arabidopsis thaliana*

**Figure S10** Development of transgenic alfalfa lines compared to WT controls.

**Figure S11** Flowering time and plant architecture measurements of transgenic alfalfa plants evaluated after 4 months of growth under LD inductive conditions.

**Figure S12** Dry weight measurements of transgenic alfalfa plants evaluated after 4 months of growth under LD inductive conditions.

**Table S1** Primers used in this study
