# Supplementary figures and images for: Improvement of alfalfa forage quality and management through the down‐regulation of MsFTa1
Source: Plant Biotechnol J. 2019 Oct 13;18(4):944–54. doi: 10.1111/pbi.13258 (PMC7061867; doi:10.1111/pbi.13258)

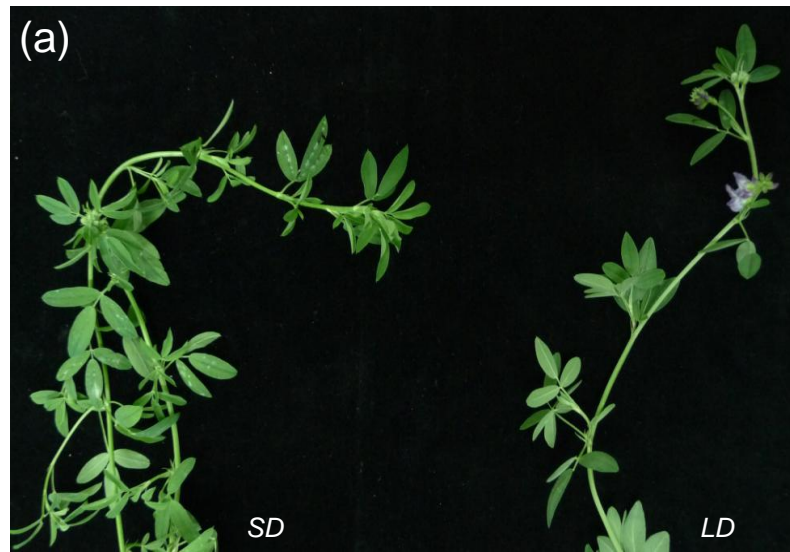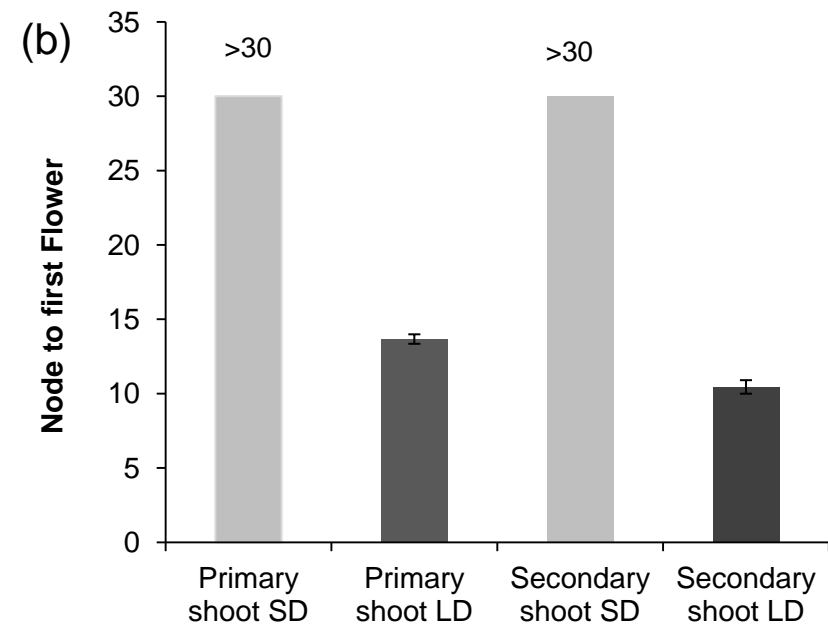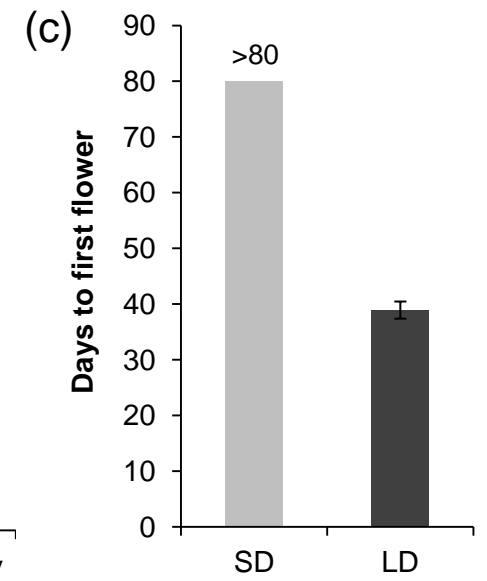

Supplement: Supplementary file 1 — Figure S1 Effect of photoperiodic induction of flowering in Medicago sativa cv Patricia. [file PBI-18-944-s001.pdf]

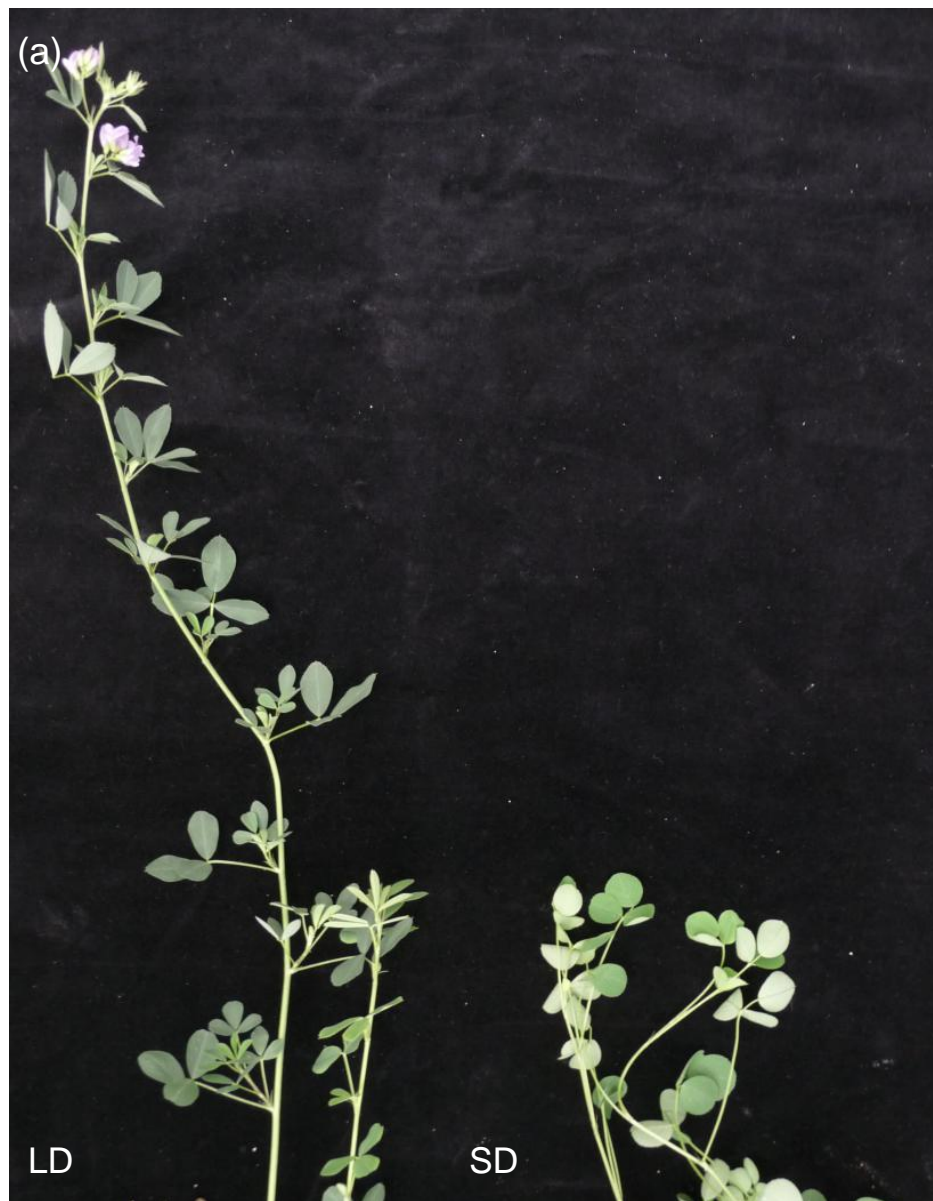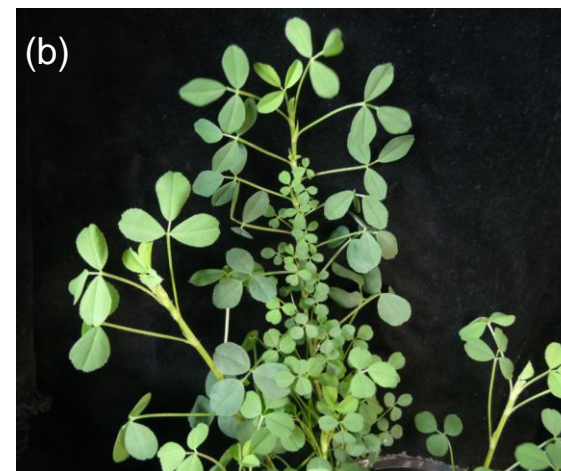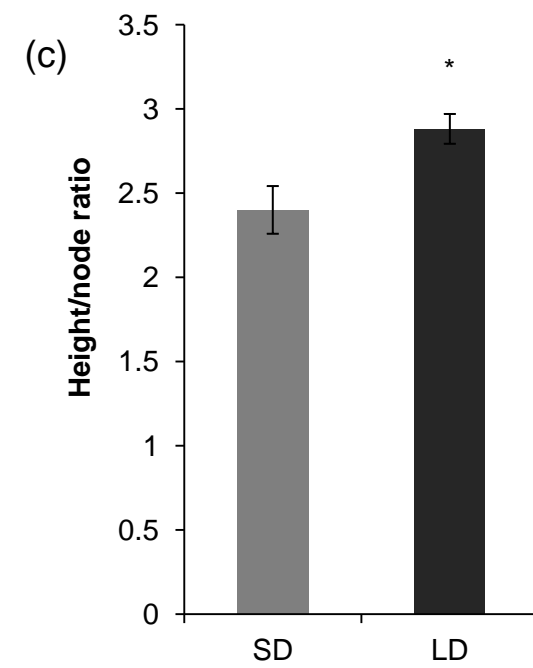

Supplement: Supplementary file 2 — Figure S2 Development and plant architecture of alfalfa plants grown under SD vs LD. [file PBI-18-944-s012.pdf]

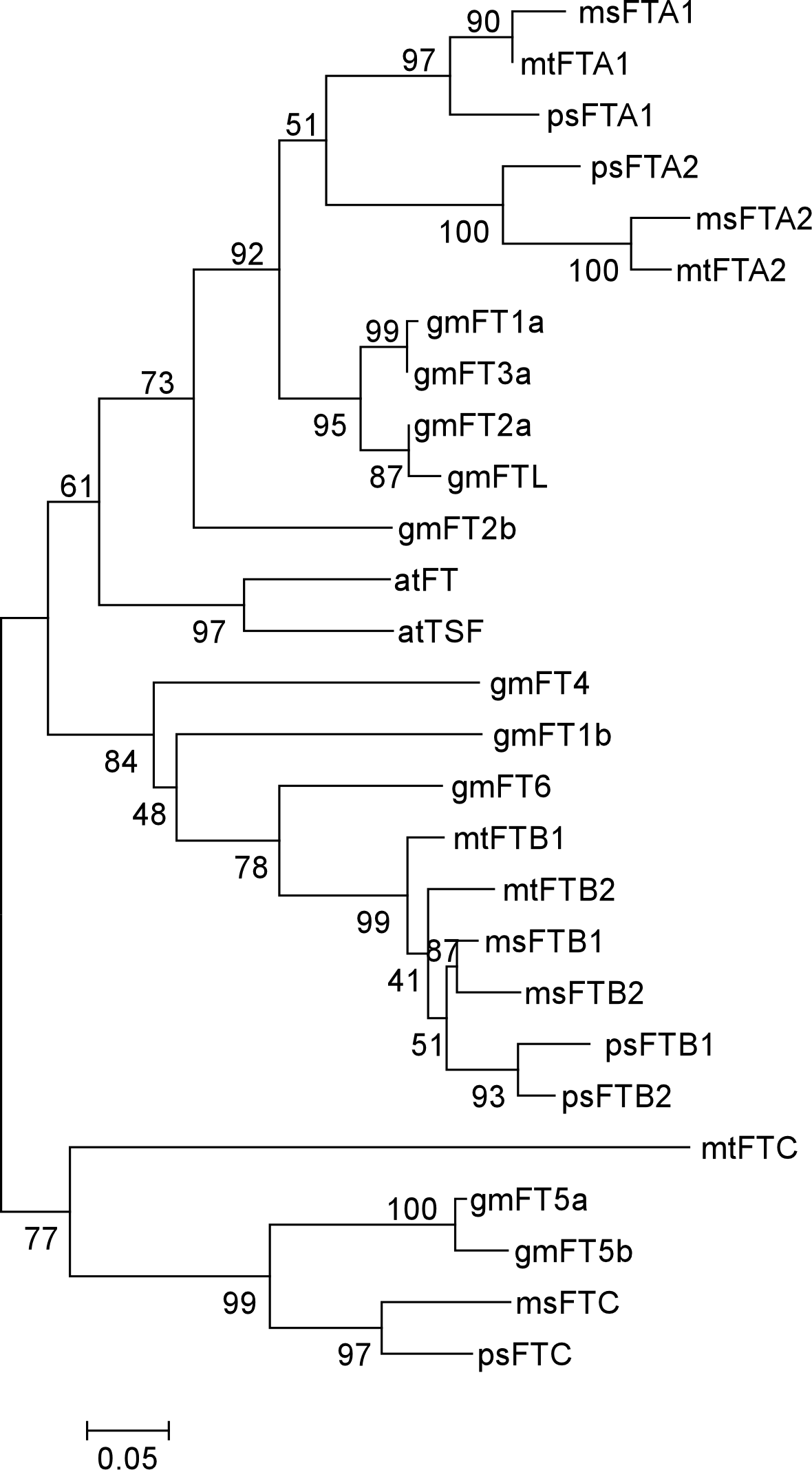

Supplement: Supplementary file 3 — Figure S3 Phylogenetic trees of proteins coded by 5 identified msFT orthologues in Medicago sativa compared to other legume orthologues. [file PBI-18-944-s011.tif]

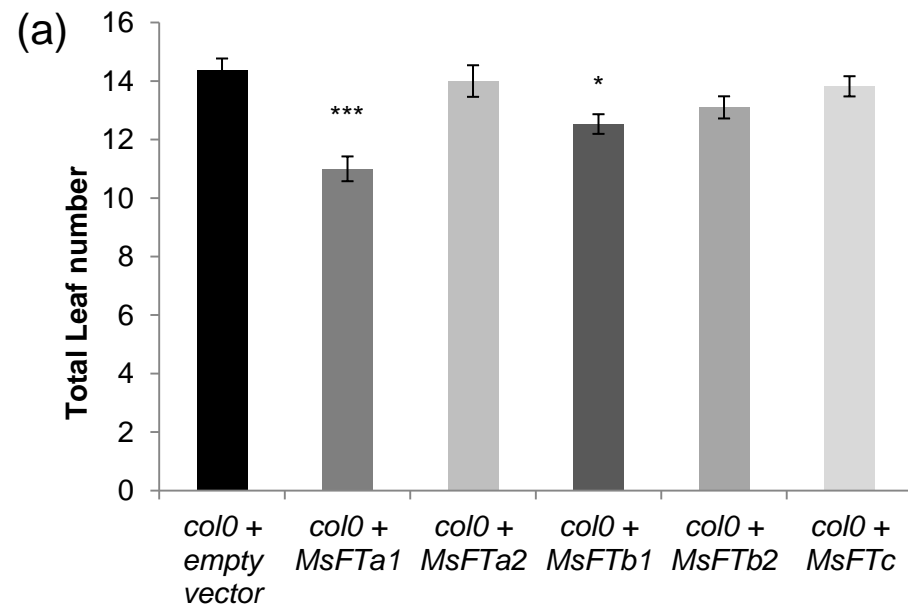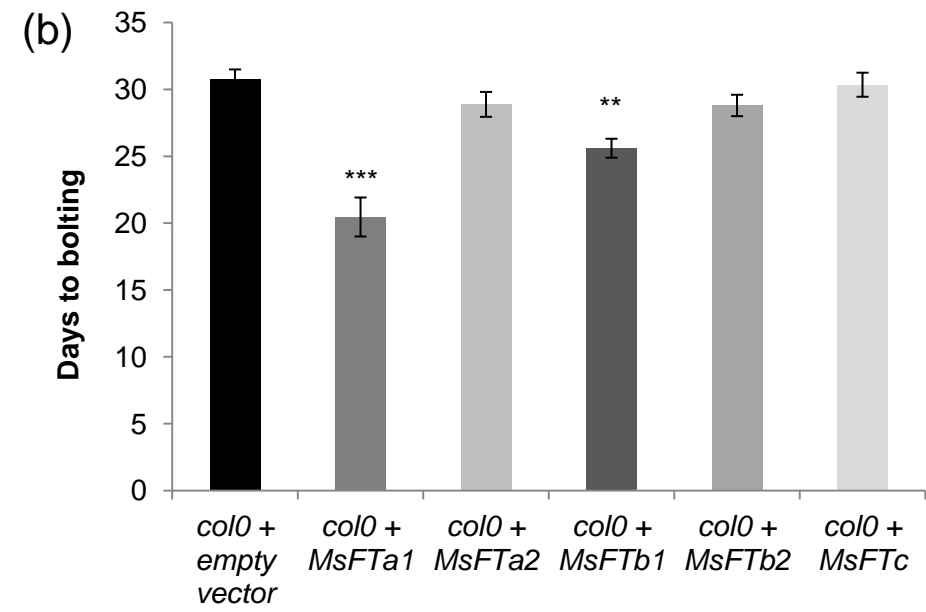

Supplement: Supplementary file 6 — Figure S6 Flowering time of transgenic WT Arabidopsis constitutively expressing msFTs orthologues. [file PBI-18-944-s002.pdf]

(a)

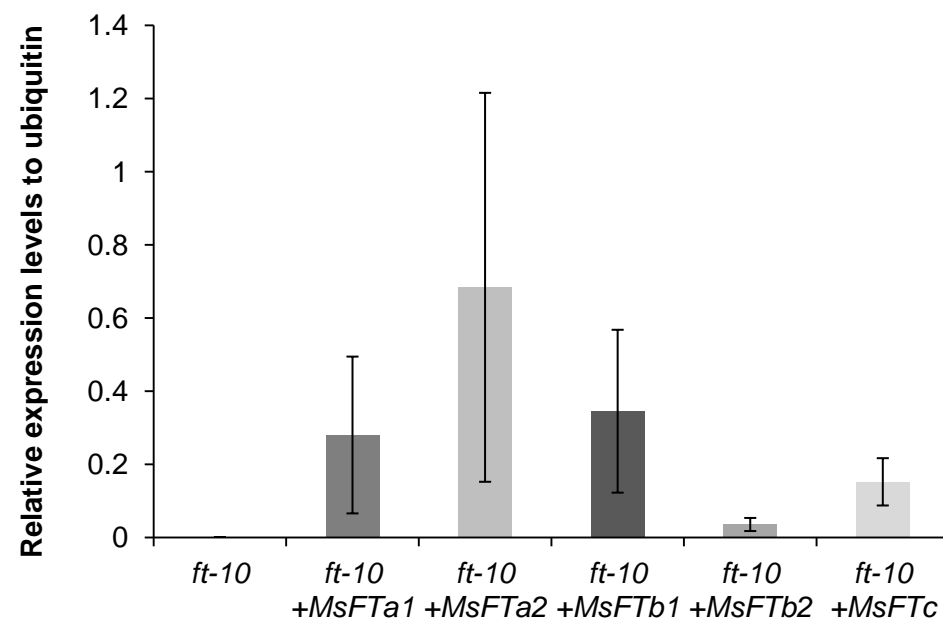

(b)

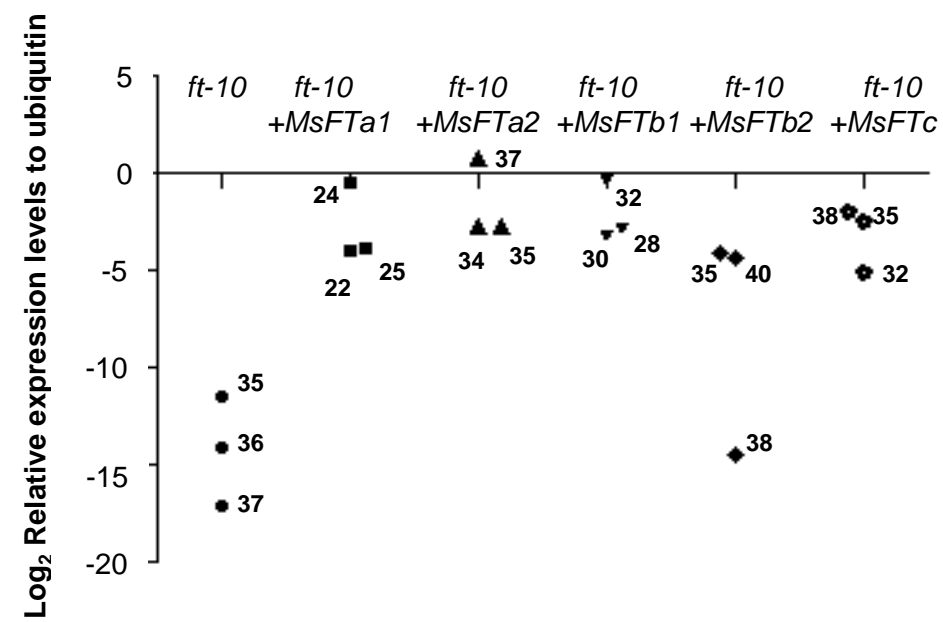

Supplement: Supplementary file 7 — Figure S7 Expression levels of alfalfa MsFTs in Arabidopsis ft‐10 background. [file PBI-18-944-s003.pdf]

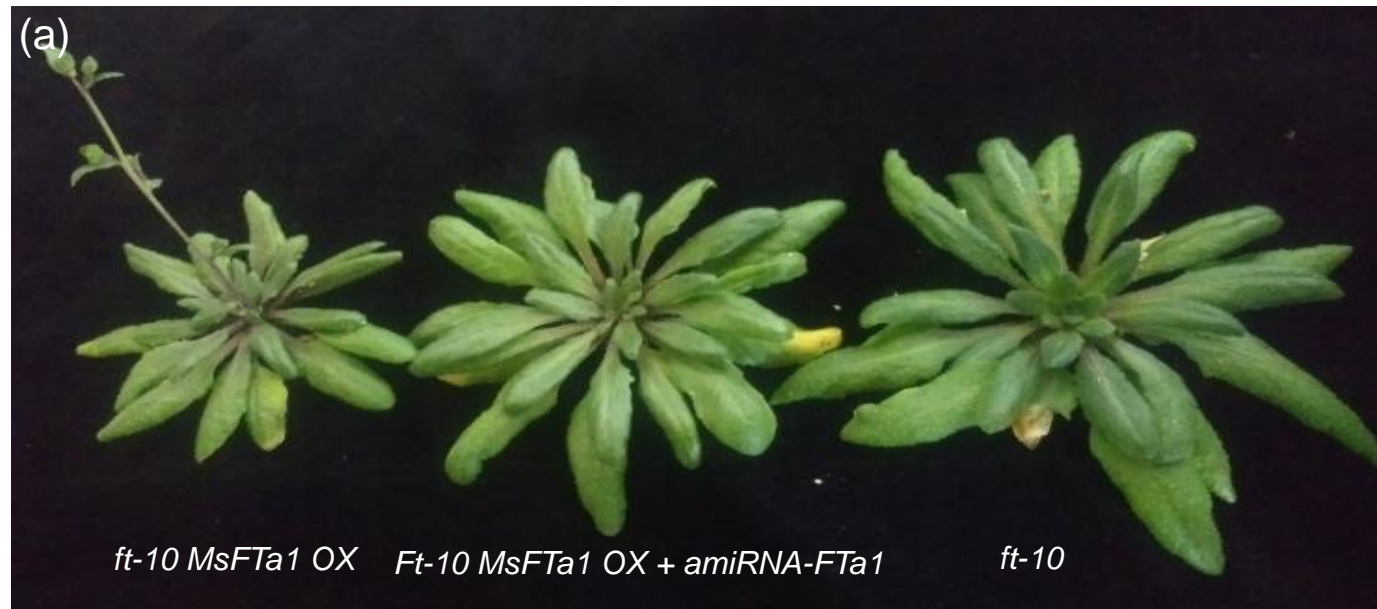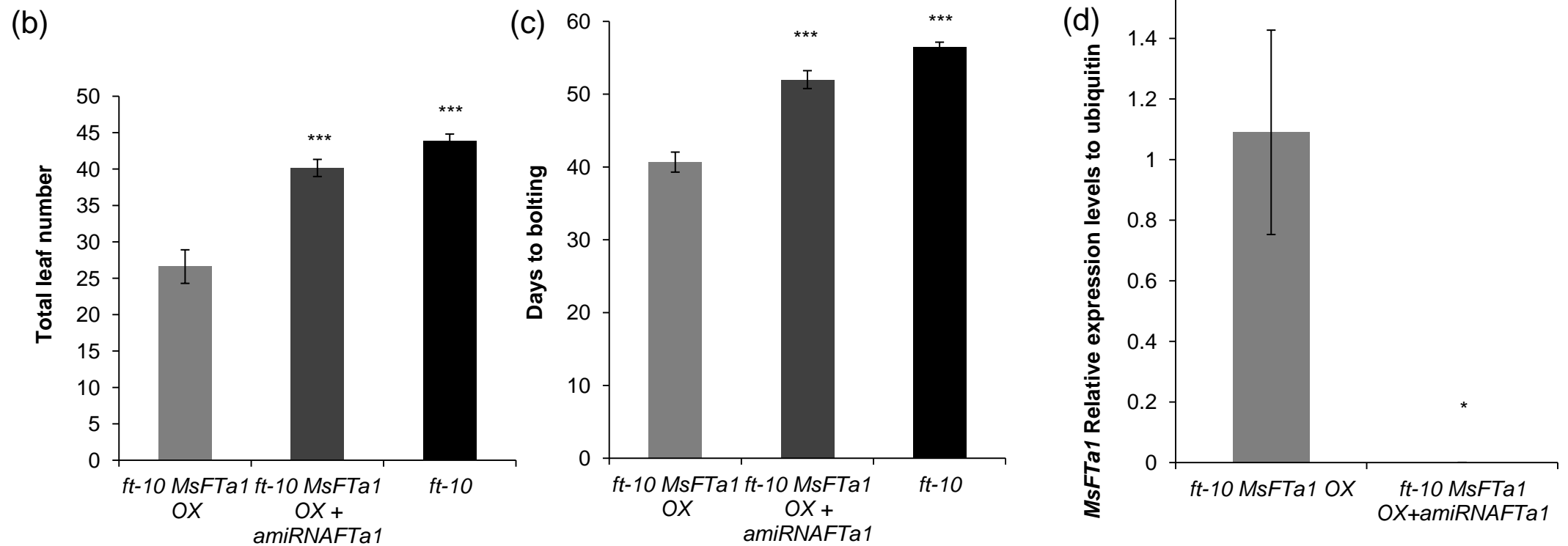

Supplement: Supplementary file 9 — Figure S9 Evaluation of amiRNA‐FTa1 in Arabidopsis thaliana [file PBI-18-944-s005.pdf]

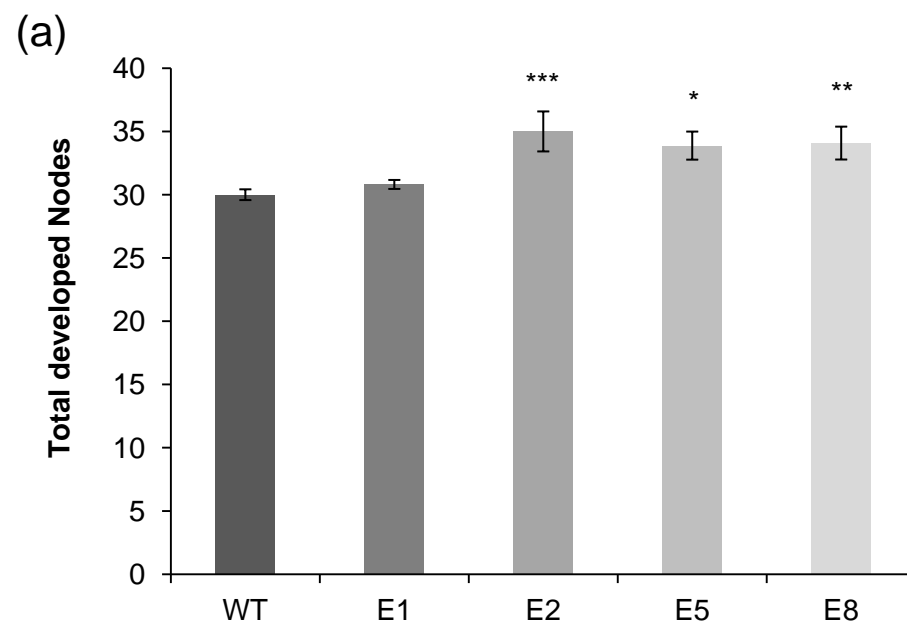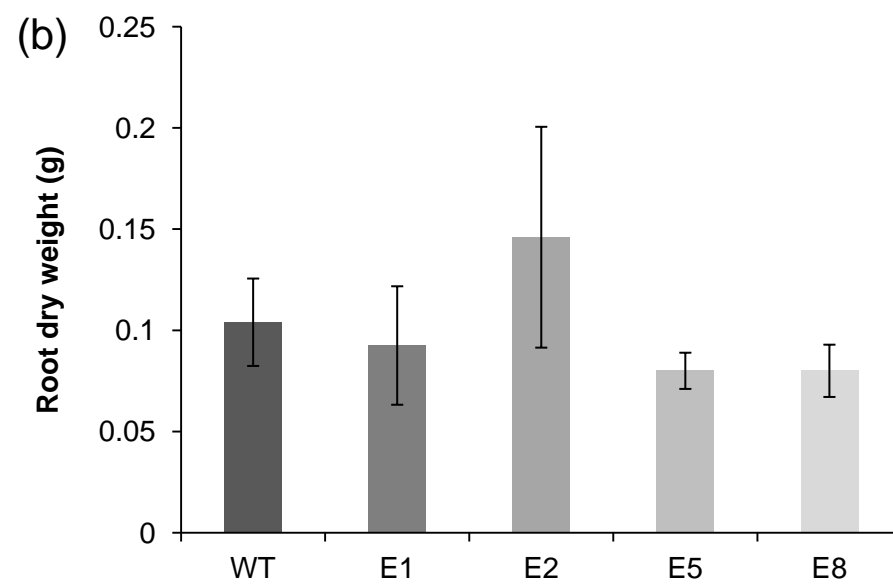

Supplement: Supplementary file 10 — Figure S10 Development of transgenic alfalfa lines compared to WT controls. [file PBI-18-944-s006.pdf]

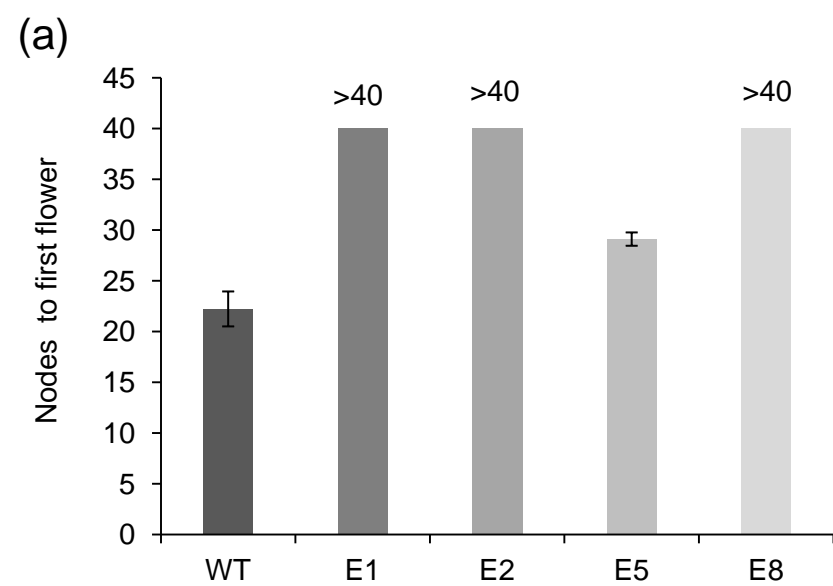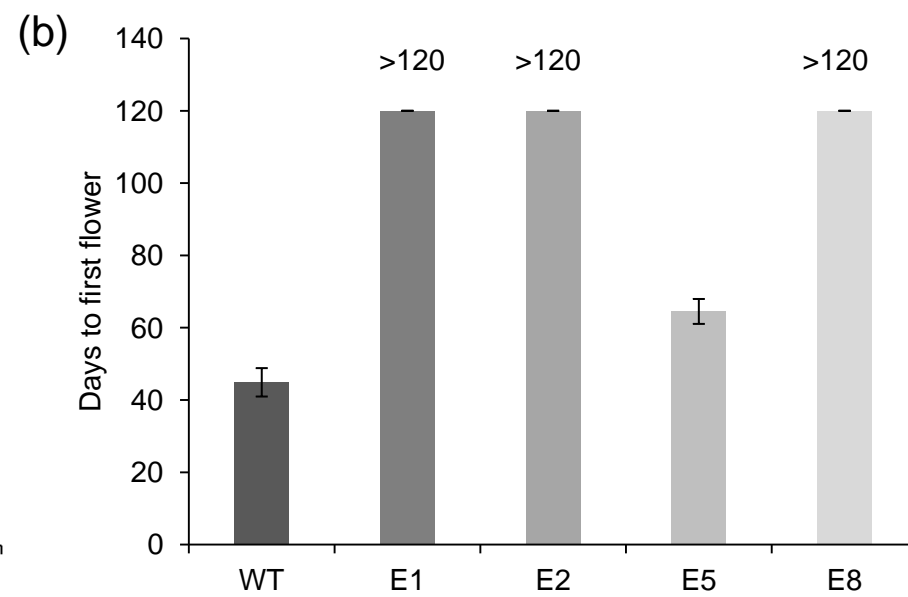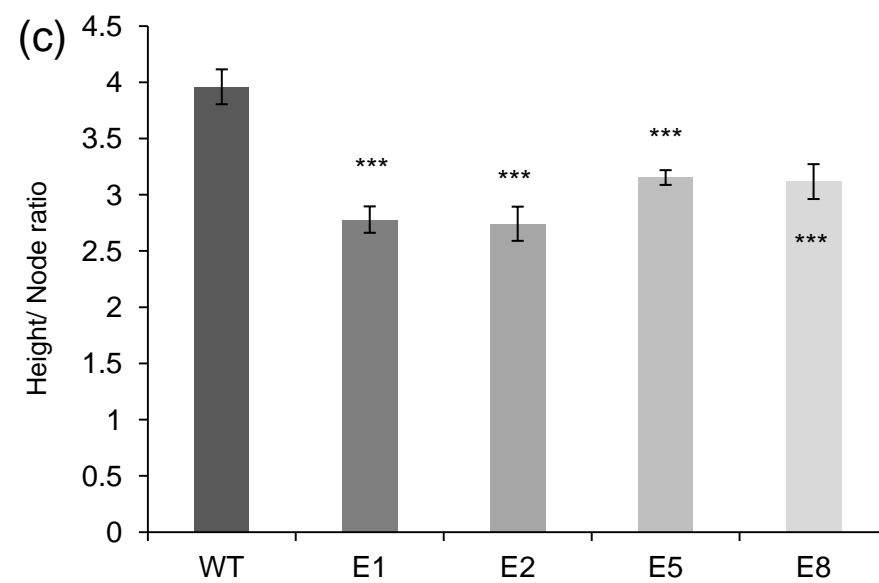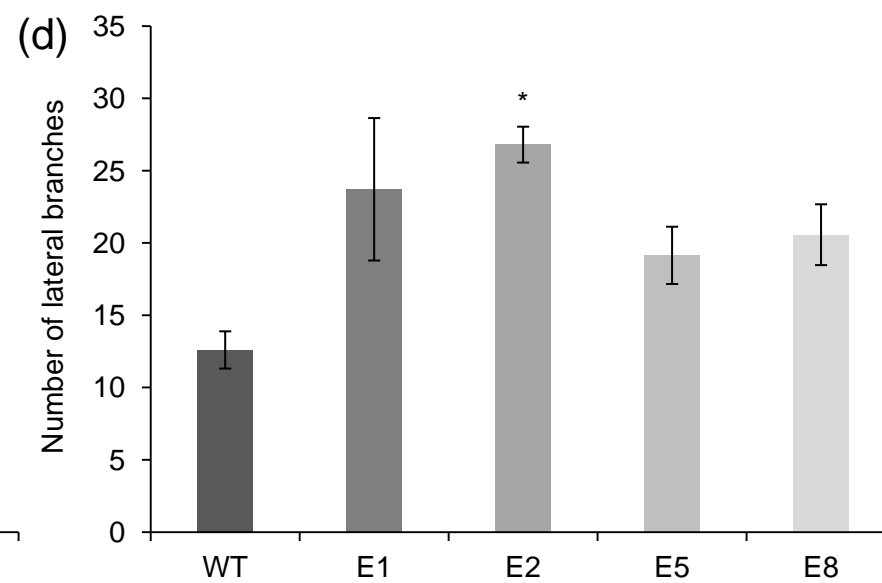

Supplement: Supplementary file 11 — Figure S11 Flowering time and plant architecture measurements of transgenic alfalfa plants evaluated after 4 months of growth under LD inductive conditions. [file PBI-18-944-s007.pdf]

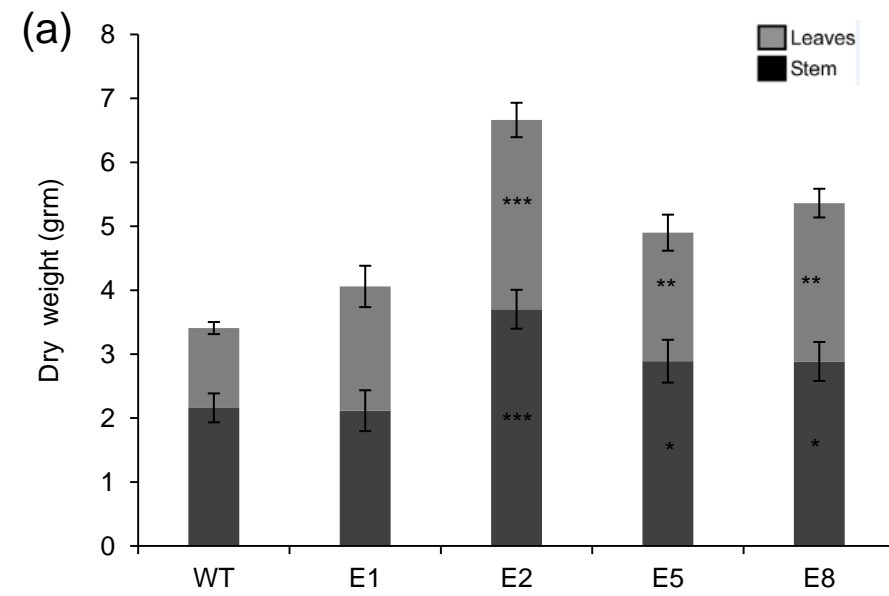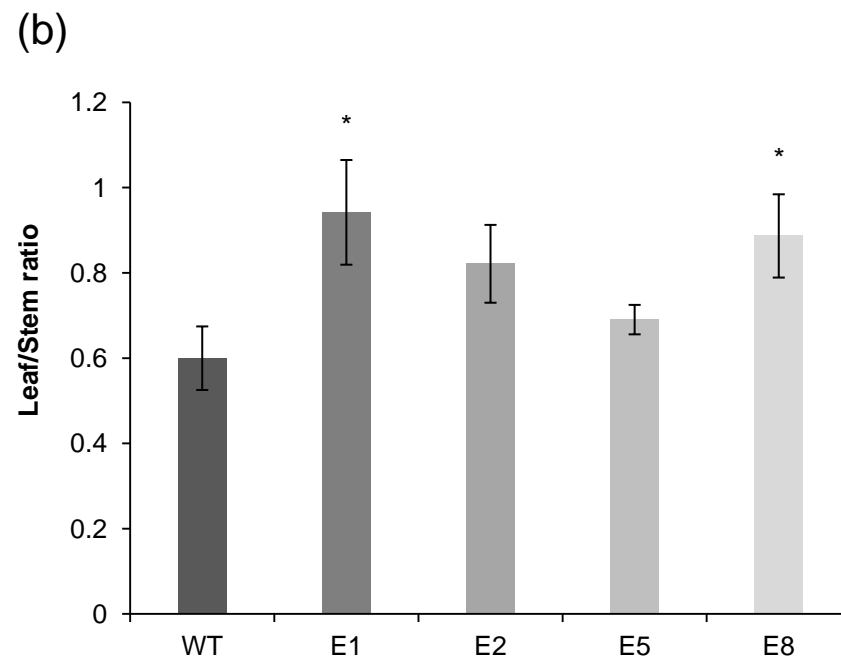

Supplement: Supplementary file 12 — Figure S12 Dry weight measurements of transgenic alfalfa plants evaluated after 4 months of growth under LD inductive conditions. [file PBI-18-944-s008.pdf]
