# Supplementary material for: Improvement of alfalfa forage quality and management through the down‐regulation of MsFTa1
Source: Plant Biotechnol J. 2019 Oct 13;18(4):944–54. doi: 10.1111/pbi.13258 (PMC7061867; doi:10.1111/pbi.13258)
Supplement: Supplementary file 4 — Figure S4 Aminoacid alignment of MsFT protein sequences including different alleles available in public databases. [file PBI-18-944-s013.pdf]

MsFTa1

```
MsFTa1          MAGSSRNPLAVGRIIGDVIDSFESSIPLRVTYGNKDVNNGCELKPSQIGNQPRVSVGGND 60
MSAD_239509     MAGSSRNPLAVGRVIGDVIDSFESSIPLRVTYGNKDVNNGCELKPSQIGNQPRVSVGGND 60
                *************:**********************************************

MsFTa1          LRNLNTLVMVDPDSPSPSNPTFKEYLHWLVTDIPGTTEVTFGNEVVNYERPRPTSGIHR 119
MSAD_239509     LRNLYTLVMVDPDSPSPSNPTFKEYLHWLVTDIPGTTEVTFGNEVVNYERPRPTSGIHR 120
                **** ******************************************************

MsFTa1          FVFVLFHQQCRQRVYAPGWRQNFNTREFAELYNLGSPVAAVFFNCQRESGSGGRTFR 176
MSAD_239509     FVFVLFHQQCRQRVYAPGWRQNFNTREFAELYNLGSPVAAVFFNCQRESGSGGRTFR 177
                *********************************************************
```

MsFTa2

```
MSAD_231541     MATGSRPNPLAVGNVIGDVLDPFESSIPLLVTYGNRTVTNGRELKPSQVANQPQVIIGVN 60
MSAD_231531     MATGSRPNPLAVGRVIGDVLDPFESSIPLLVTYGNRTVTNGRQLKPSQVANQPQVIIGVN 60
MsFTa2          MATGSRPNPLAVGRVIGDVLDPFESSIPLLVTYGNRTVTNGRELKPSQVANQPQVIIGVN 60
MSAD_239702     MATGGRPNPLAVGRVIGDVLDPFESSIPLLVTYGNRTVTNGRELKPSQVANQPQVIIGVN 60
                ****.********.****************************:*****************

MSAD_231541     DPTTFYTLVLVDPDAPSPSYPSHREYLHWMVTDIPSTNAASFGNEVVSYEKPRPNLGIH 119
MSAD_231531     DPTTFYTLVLVDPDAPSPSYPSFREYLHWMVTDIPATNAASFGNEVVSYEKPRPNLGIH 119
MsFTa2          DPTTFYTLVLVDPDAPSPSYPSFREYLHWMVTDIPATNAASFGNEVVSYEKPRPNLGIH 119
MSAD_239702     DPTTFYTLVLVDPDAPSPSYPSFREYLHWMVTDIPATNAASFGNEVVSYEKPRPNLGIH 120
                **********************.************:***********************

MSAD_231541     RYVFVLLRQQCSQRVYAPGWRQNFNTREFTEFYDLGSPVAAVFFNCQRENGSGGRTFR 176
MSAD_231531     RYVFVLLRQQCSQRVYAPGWRQNFSTREFTEFYDLGSPVAAVFFNCQRENGSGGRTFR 177
MsFTa2          RYVFVLLRQRCSQRVYAPGWRQNFNTREFIEFYDLGSPVAAVFFNCQRETGSGGRTFR 177
MSAD_239702     RYVFVLLRQRCSQRVYAPGWRQNFNTREFIEFYDLGSPVAAVFFNCQRETGSGGRTFR 178
                *********:**************.**** *******************.********
```

MsFTb1

```
MSAD_303499     MHINSMNPLVVGGVIGEVLDPFTNSVSLRVVYGNNKEVINSGELKPSQIVNPPSVQVGGN 60
MsFTb1          MRIKSLNPLVVCGVIGDVLDPFTNSVSLRVVYDNNKEVINSGELKPSQIVNPPRVQVGGN 60
MSAD_249423     MPIRSMNPLVVCGVIGDVLDPFTNSVSLRVVYNNNKEVNNGGELKPSQIVNQPRVQVGGH 60
MSAD_291393     MPIKSMNPLVVCGVIGDVLDPFTNSVSLRVVYNNNKEVNNGGELKPSQIVNQPRVQVGGH 60
                * *.*:***** ****:***************.***** *.********** * *****:

MSAD_303499     DLRTLYTLVMVNPDAPSPTDPNMREYLYWMVTNIPATTGTTFGQEIVSYESPRPTSGI 120
MsFTb1          DLRTLYTLVMVNPDAPSPSNPTMREYLYWMVTNIPATTGTTFGEEIVSYESPRPTSGI 118
MSAD_249423     DLRTLYTLVMVDPDGPSPSNPNMREYLHWMVTNIPATTGTTFGEEIVSYESPRPTSGI 118
MSAD_291393     DLRTLYTLVMVDPDGPSPSNPNMREYLHWMVTNIPATTGTTFGEEIVSYESPRPTSGI 119
                ***********:**.***::*.*****:***************:**************

MSAD_303499     QRVIFVLFRQPCRHTMLAPRRRQNFITRDFAEVYNLGLPVAALYFNCQRENGSGGRRLII 180
MsFTb1          HRVIFVLFRQPCRLTILAPGWRQNFITRDFAEVYNLGLPVAALYFNCQRENGSGGRRLII 178
MSAD_249423     HRVIFVLFRQPCRLTILAPGWRQNFITRDFAEVYNLGLPVAALYFNCQRENGSGGRRLII 178
```

```
MSAD_291393       HRVIFVLFRQPCRLTILAPGWRQNFITRDFAEVYNLGLPVAALYFNCQRENGSGGRRLII 179
                  :************ *:***  ***************************************
```

MsFTb2

```
MsFTb2            MRIKSMNPLVVCGVIGDVLDPFTNSVSLRVVYDNNKEVINSGELEPSQIVNPPRVQVGGN 60
MSAD_291397       MRIKSMNPLVVCGVIGDVLDPFTNSVSLRVVYDNNKEVINSGELKPSQIVNPPRVQVGGN 60
MSAD_303504       MRIKSMNSLVVGGVIGEVLDPFTNSVSLRVVYENNKEVINGGELKPSQIVNPPRVQVGGN 60
MSAD_291383       MRIKSMNSLVVGGVIGEVLDPFTNSVSFRVVYENNKEVINGGELKPSQIVNPPRVQVGGN 60
                  ******* *** ****:**********:****:*******.***:***************

MsFTb2            DLRTLYTLVMVNPDAPSPSNPTMREYLYWMVTNIPATTGTTFGQEIVSYESPRPT 115
MSAD_291397       DLRTLYTLVMVNPDAPSPSNPTMREYLYWMVTNIPATTGTTFGQEIVSYESPRPT 116
MSAD_303504       DLRTLYTLVMVNPDAPSPTDPNMREYLYRMVTNIPATTGTTFGQEIVSYENPRPT 118
MSAD_291383       DLRTLHTLVMVNPDAPSPTDPNMREYLYRMVTNIPATTGTTFGQEIVSYENPRPT 117
                  *****:************::*.****** *********************.****

MsFTb2            SGIQRVIFVLFRQPCRHNILAPGWRQNFNTRDFVEVHNLGLPVAALYFNCQRENGSGGRR 175
MSAD_291397       SGIQRVIFVLFRQPCRHNILAPGWRQNFNTRDFAEVYNLGLPVAALYFNCQRENGSGGRR 176
MSAD_303504       RGIHRVIFVLFRQPCRHTILAPRSRQNFITRDFAEVYNLGLPVAALYFNCQRESGSGGRR 178
MSAD_291383       RGIHRVIFVLFKQPCRHTILAPRSRQNFITRDFAEVYNLGLPVAALYFNCQRESGSGGRR 177
                   **:*******:*****.****  **** ****.**:****************.******

MsFTb2            MVM   178
MSAD_291397       MVM   179
MSAD_303504       LTI   181
MSAD_291383       LTI   180
                  :.:
```

MsFTc

```
MsFTc             MPQNLVDPLGVIGDVLNPFTNSVSLSAIINNREISNGCLMKPSQLVNRPRVNVGGDDLRT 60
MSAD_239703       MPQNLADPLGVIGDVLNPFTNSVSLSAIINNREISNGCLMKPSQLVNRPRVNVGGDDLRT 60
                  *****.******************************************************

MsFTc             FYTMVMVDADAPSPSNPFLKGYLHWMVTDIPATTSASFGKEVVFYESPKPSAGIHRFVI 119
MSAD_239703       FYTMVMVDADAPSPSNPFLKEYLHWMVTDIPATTSASFGKEVVFYESPKPSAGIHRFVI 120
                  ******************** **************************************

MsFTc             ALFKQLGRDTVFAPDWRHNFNTMSFAEINNLVIVASVYFNCQRERGCGGRRC 171
MSAD_239703       ALFKQLGRDTVFAPDWRHNFNTMSFAEINNLVIVASVYFNCQRERGCGGRRC 172
                  ****************************************************
```
