# Supplementary material for: Improvement of alfalfa forage quality and management through the down‐regulation of MsFTa1
Source: Plant Biotechnol J. 2019 Oct 13;18(4):944–54. doi: 10.1111/pbi.13258 (PMC7061867; doi:10.1111/pbi.13258)
Supplement: Supplementary file 5 — Figure S5 Aminoacid alignment of functionally chacterised FTc orthologues in legumes. [file PBI-18-944-s014.pdf]

```
MsFTC     MPQNLVDPLGVIGDVLNPFTNSVSLSAIINNREISNGCLMKPSQLVNRPRVNVGGDDLRT     60
MtFTc     MPQNLVDPLGVIGDVLSPFTNSVSLSALINNREISNGCIMKPSQLVNRPRVNVGGDDLRT     60
PsFTc     MPQNLVDPLGVIGDVLSPFTNSVSLSALINNREISNGCIMKPSQLVNRPRVNVGGDDLRT     60
          ****************.**********:**********:*********************

MsFTC     FYTMVMVDADAPSPSNPFLKGYLHWMVTDIPATTSASFGKEVVFYESPKPSAGIHRFVIA     120
MtFTc     FYTMVMVDADAPSPSNPFLKEYLHWMVTDIPATTSASFGKEVVFYESPKPSAGIHRFVIA     120
PsFTc     FYTMVMVDADAPSPSNPFLKEYLHWMVTDIPATTSASFGKEVVFYESPKPSAGIHRFVIA     120
          ******************** ***************************************

MsFTC     LFKQLGRDTVFAPDWRHNFNTMSFAEINNLVIVASVYFNCQRERGCGGRRC     171
MtFTc     LFKQLGRDTVFAPDWRHNFNTTNFAEINNLVIVASVYFNCQRERGCGGRRC     171
PsFTc     LFKQLGRDTVFAPDWRHNFNTTNFAEINNLVIVASVYFNCQRERGCGGRRC     171
          ********************* .****************************
```
