# Supplementary material for: Improvement of alfalfa forage quality and management through the down‐regulation of MsFTa1
Source: Plant Biotechnol J. 2019 Oct 13;18(4):944–54. doi: 10.1111/pbi.13258 (PMC7061867; doi:10.1111/pbi.13258)
Supplement: Supplementary file 8 — Figure S8 Secondary structure and sequence of amiRNA‐FTa1 [file PBI-18-944-s004.pdf]

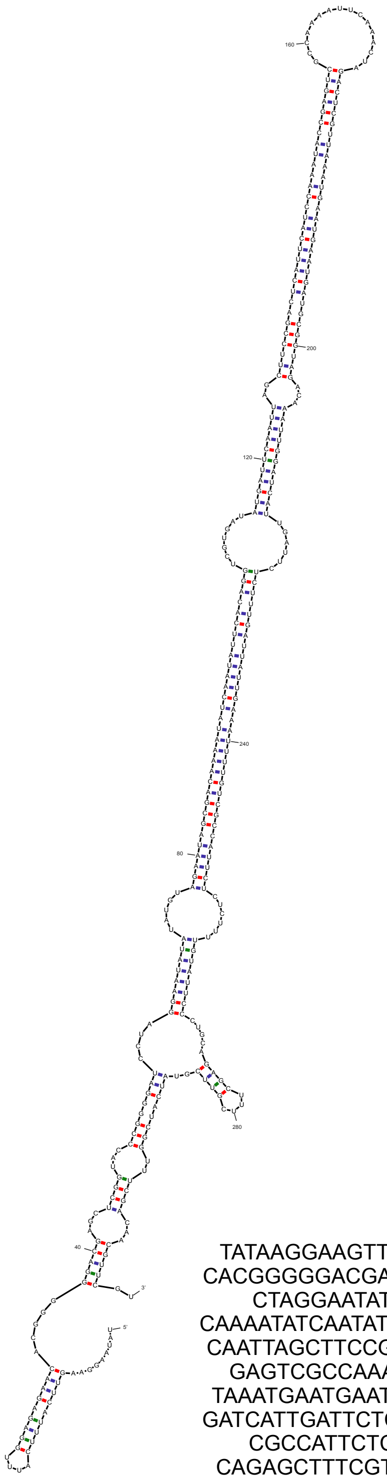

TATAAGGAAGTTCATTTCATTTGGAGAGAA-
CACGGGGGACGAGCTCGGTACCCGGGGATC-
CTAGGAATATATATGTAGAATAGCGA-
CAAAATATCAATATTCACAGGTCGTGATATGATT
CAATTAGCTTCCGACTCATTCATCCAAATACC-
GAGTCGCCAAAATTCAAACTAGACTCGT-
TAAATGAATGAATGATGCGGTAGACAAATTG-
GATCATTGATTCTCTTTGATTATTGAAATTTTGT
CGCCATTCTCTCTTTTGTATTCCCTG-
CAGAGCTTTCGTTCGTATCATCGGTTTCGA-
CAACGTTCGT
