# Supplementary material for: Improvement of alfalfa forage quality and management through the down‐regulation of MsFTa1
Source: Plant Biotechnol J. 2019 Oct 13;18(4):944–54. doi: 10.1111/pbi.13258 (PMC7061867; doi:10.1111/pbi.13258)
Supplement: Supplementary file 14 — Supplementary Legends [file PBI-18-944-s010.docx]

Figure S1: Effect of photoperiodic induction of flowering in *Medicago sativa* cv Patricia (a) Phenotype of plants at phenological equivalent stages growth under SD and LD. (b) Flowering time measured as nodes to first flower for the primary and secondary stems and (c) Days to first flower for the primary stem under SD and LD. Bars represent the means + SE of 15 individual grown plants per condition.

Figure S2: Development and plant architecture of alfalfa plants grown under SD vs LD. (a) Phenotype of plants grown under LD vs plant grown under SD for one month. (b) Phenotype of overly grown trifoliates leaves for plants grown under SD for extended period of times. (c) Height to node number of plants grown under SD vs LD. Bars represent the means + SE of 15 individual grown plants per condition. Results were analyzed by a T-student test. Asterisks represent different levels of significance. (p <0.05=*)

Figure S3: Phylogenetic trees of proteins coded by *5* identified *msFT* orthologues in *Medicago sativa* compared to other legume orthologues. Different initials stand for: ms (*Medicago sativa*), mt (*Medicago truncatula*), gm (*Glycine max*), ps (*Pisum sativum*), cc (*Cajanus Cajun*), lj (*Lotus japonicus*, at *Arabidopsis* thaliana. All trees were developed using the Maximum likelihood method and a bootstrapping of 1000. Bootstraping values are indicated at each branch.

Figure S4: Aminoacid alignment of MsFT protein sequences including different alleles available in public databases. Gray-shaded sequences remarks the alleles of MsFTs characterized in this paper. Tyr 85 and Gln 140 critical residues (or their equivalent position in each sequence) are highlighted in green and aminoacids belonging to segment B are underlined and highlighted in bold letters.

Figure S5: Aminoacid alignment of functionally chacterised FTc homologues in legumes. Gray-shaded sequences remarks the allele of MsFTc characterized in this paper. Putative critical differential residues are highlighted in red and aminoacids belonging to segment B are underlined and highlighted in bold letters.

Figure S6: Flowering time of transgenic WT Arabidopsis constitutively expressing *msFTs* orthologues. Flowering time was measured as both (a) total leaf number and (b) days to bolting. Bars represent the means + SE of 10-15 individual T1 selected plants per construct. Results were analysed by a one way ANOVA with posterior Dunnet test. Asterisks represent different levels of significance. (p <0.05=*, p< 0.01=**, p<0.001=***).

Figure S7: Expression levels of alfalfa *MsFTs* in Arabidopsis *ft-10* background. mRNA levels of the *MsFTs* were quantificated by qPCR, relative to *UBQ10* as a housekeeping control expressed represented as both mean relative expression levels (a) and log2 of expression levels as a scatter plot (b). RNA was extracted from leaves of 3 weeks-old T2 plants. Bars represent the means + SE of 3 independent T2 populations per construct. The value next to each data point in (b) represents the total leaf number of the T1 lines these T2 populations descended from. Results were analyzed by a one way ANOVA with posterior Dunnet test. Asterisks represent different levels of significance. (p <0.05=*, p< 0.01=**, p<0.001=***).

Figure S8: Secondary structure and sequence for precursor of *amiRNA-FTa1*

Figure S9: Evaluation of *amiRNA-FTa1* in *Arabidopsis thaliana* (a) Flowering phenotype of *ft-10 MsFTa1*OX T3 lines compared to *ft-10 MsFTa1*OX *amiRNA-MsFTa1* and *ft-10*. (b) Flowering time was measured as total leaf number and (c) Total days to first flower. (d) Relative expression levels of *MsFTa1* in *MsFTa1* OX lines compared to silenced Arabidopsis lines. All plants were grown at 23 ºC under a LD photoperiod. Bars represent the means + SE of 3 biological replicates for qPCR experiments and 15 T1 grown plants per condition for flowering assays. Results were analyzed by a one way ANOVA with posterior Dunnet test for flowering time and T-student tests for qPCR. Asterisks represent different levels of significance. (p <0.05=*, p< 0.01=**, p<0.001=***).

Figure S10: Development of transgenic alfalfa lines compared to WT controls. (a) Total developed nodes of transgenic alfalfa plants evaluated after 2 and a half months of growth under LD flowering inductive conditions. (b) Dry weight of root tissue of transgenic alfalfa plants and WT controls evaluated after 1 and a half months. Bars represent the means + SE of 8-10 individual grown clonal plants per line per condition for flowering assays and 5 individual grown plants for root development assays. Results were analysed by a one way ANOVA with posterior Dunnet test. Asterisks represent different levels of significance. (p <0.05=*, p< 0.01=**, p<0.001=***).

Figure S11: Flowering time and plant architecture measurements of transgenic alfalfa plants evaluated in extended time assays after 4 months of growth under LD inductive conditions. (a) Flowering time measured as both nodes to first flower and (b) Days to first flower. (c) Height / node ratio and (d) number of lateral branches. For flowering assays, days and nodes to flower were counted until the moment of each line flowering or at end of the experiment. Bars represent the means + SE of 5-10 individual grown clonal plants per line. Results were analyzed by a one way ANOVA with posterior Dunnet test. Asterisks represent different levels of significance. (p <0.05=*, p< 0.01=**, p<0.001=***).

Figure S12: Dry weight measurements of transgenic alfalfa plants evaluated after 4 months of growth under LD inductive conditions. (a) Total dry weight partitioned as leaf dry weight (light grey bars) and stem dry weight (dark grey bars) and (b) Leaf / stem ratio for 4 transgenic lines (E1, E2, E5, E8) overexpressing *amiRNA-FTa1* compared to WT regenerated controls. Bars represent the means + SE of 5-10 individual grown clonal plants per line. Results were analyzed by a one way ANOVA with posterior Dunnet test. Asterisks represent different levels of significance. (p <0.05=*, p< 0.01=**, p<0.001=***).

**Table S1:** **Primers used in this study**
